# Supplementary material for: Intronic miR-6741-3p targets the oncogene SRSF3: Implications for oral squamous cell carcinoma pathogenesis
Source: PLoS One. 2024 May 23;19(5):e0296565. doi: 10.1371/journal.pone.0296565 (PMC11115324; doi:10.1371/journal.pone.0296565)
Supplement: S1 Fig — (PDF) [file pone.0296565.s001.pdf]

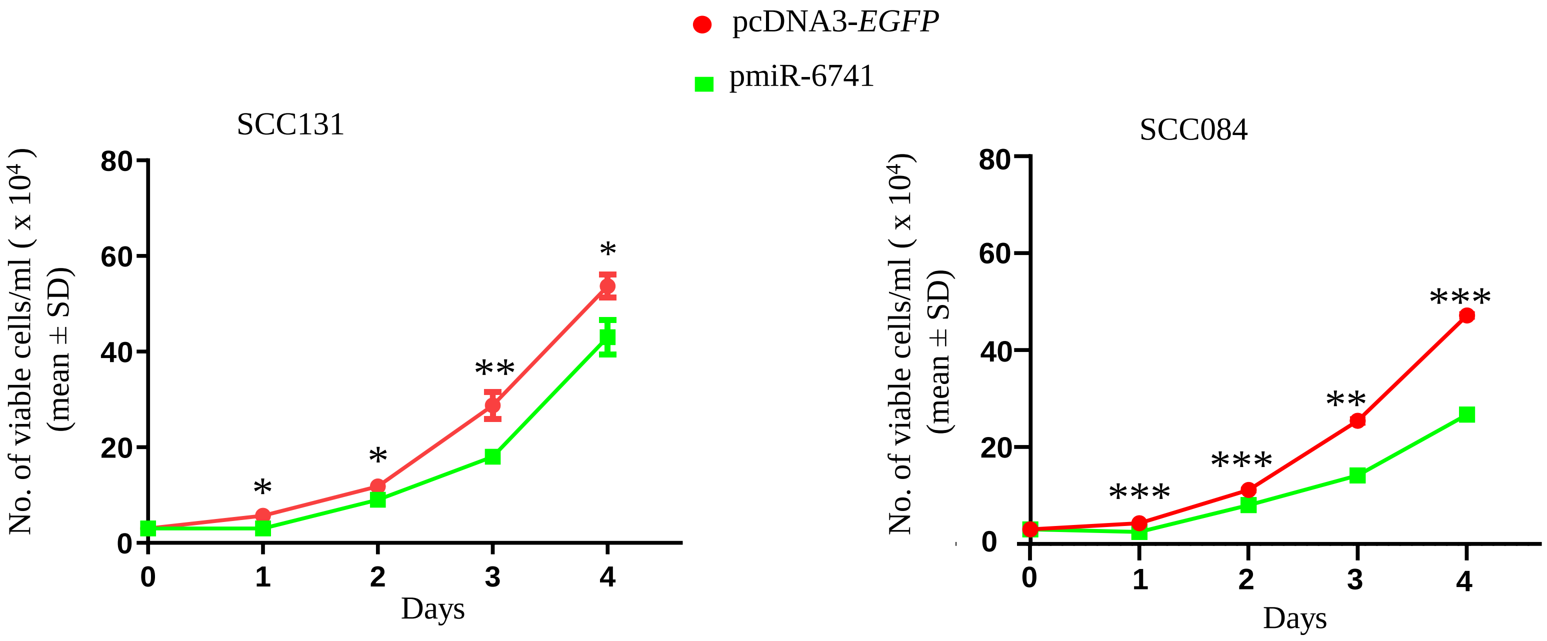

S1 Fig. miR-6741-3p decreases the proliferation of SCC131 and SCC084 cells. Trypan blue dye exclusion assay revealed that transient overexpression of miR-6741-3p using the pmiR-6741 construct decreases the proliferation of both SCC131 and SCC084 cells compared to the vector control transfected cells. Each data point is an average of 3 biological replicates.
