# Supplementary material for: Intronic miR-6741-3p targets the oncogene SRSF3: Implications for oral squamous cell carcinoma pathogenesis
Source: PLoS One. 2024 May 23;19(5):e0296565. doi: 10.1371/journal.pone.0296565 (PMC11115324; doi:10.1371/journal.pone.0296565)
Supplement: S2 Fig — (PDF) [file pone.0296565.s002.pdf]

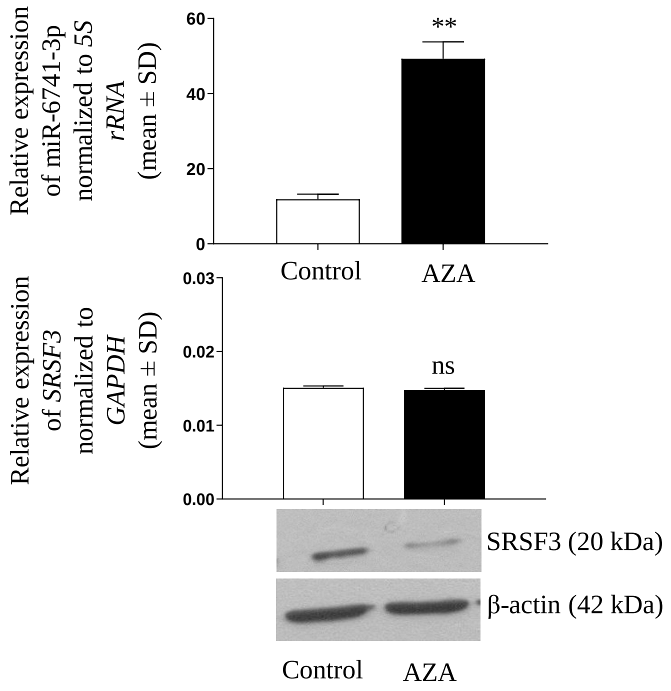

S2 Fig. 5-Azacytidine treatment of SCC131 cells upregulates miR-6741-3p expression and downregulates SRSF3. Following 5-Azacytidine treatment, with an increase in miR-6741-3p expression, there is a concomitant decrease in the SRSF3 protein level with no change in its transcript level. Each bar for qRT-PCR is an average of 2 technical replicates. β-actin was used as a loading control.
