# Supplementary material for: Intronic miR-6741-3p targets the oncogene SRSF3: Implications for oral squamous cell carcinoma pathogenesis
Source: PLoS One. 2024 May 23;19(5):e0296565. doi: 10.1371/journal.pone.0296565 (PMC11115324; doi:10.1371/journal.pone.0296565)
Supplement: S3 Fig — (PDF) [file pone.0296565.s003.pdf]

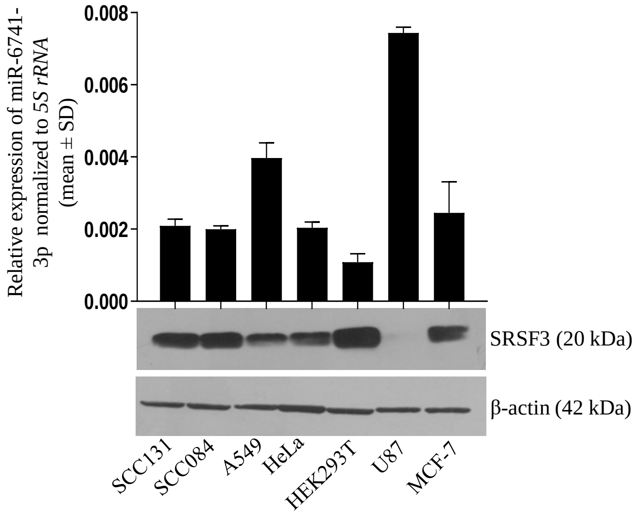

S3 Fig. Expression analysis of miR-6741-3p and SRSF3 in cell lines. In general, an inverse correlation between the expression of miR-6741-3p and SRSF3 was observed across the cell lines. Each qRT-PCR data is an average of 2 technical replicates.
