# Supplementary material for: Intronic miR-6741-3p targets the oncogene SRSF3: Implications for oral squamous cell carcinoma pathogenesis
Source: PLoS One. 2024 May 23;19(5):e0296565. doi: 10.1371/journal.pone.0296565 (PMC11115324; doi:10.1371/journal.pone.0296565)
Supplement: S4 Fig — (PDF) [file pone.0296565.s004.pdf]

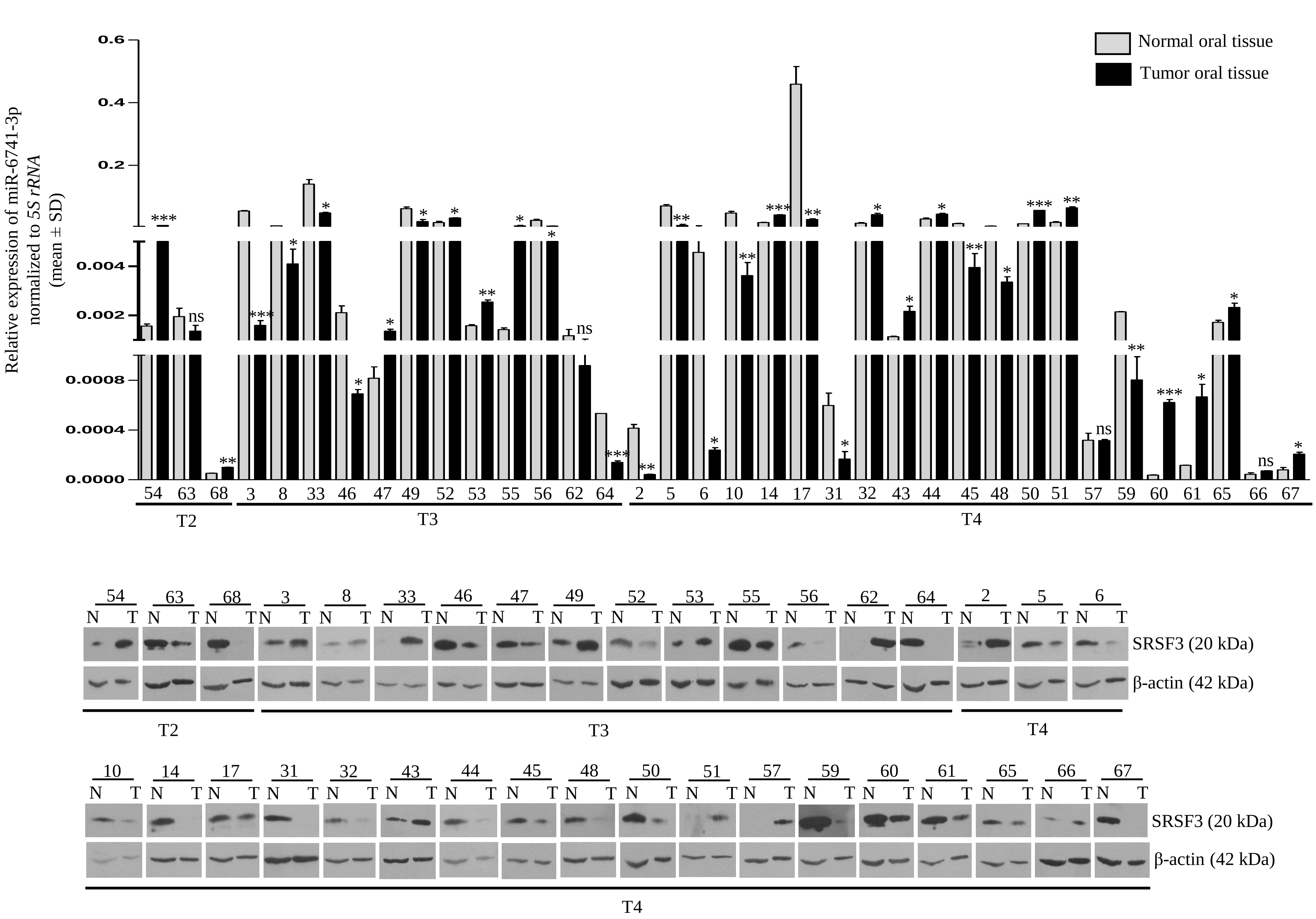

S4 Fig. Expression analysis of miR-6741-3p and SRSF3 in OSCC patient samples. The relative expression of miR-6741-3p is shown in the upper panel, and of *SRSF3* in the lower panel. N and T represent adjacent normal oral and tumor tissue samples respectively. Numbers along X-axis and on the top of the immunoblots represent patient numbers. T2, T3 and T4 represent different stages of OSCC. Each bar in qRT-PCR data is an average of 2 technical replicates. Note, expression in OSCC tumor tissues relative to matched normal oral tissues; miR-6741-3p downregulation: patient no. 3, 8, 33, 46, 49, 56, 64, 2, 5, 6, 10, 17, 31, 45, 48, and 59; miR-6741-3p upregulation: patient no. 54, 68, 47, 52, 53, 55, 14, 32, 43, 44, 50, 51, 60, 61, 65, and 67; miR-6741-3p no change in expression: patient no. 63, 62, 57, and 66; *SRSF3* upregulation: patient no. 54, 3, 8, 33, 49, 53, 62, 2, 43, 51, 57, and 66; *SRSF3* downregulation: patient no. 63, 68, 46, 47, 52, 55, 56, 64, 5, 6, 10, 14, 17, 31, 32, 44, 45, 48, 50, 59, 60, 61, 65, and 67.
