# Supplementary material for: Intronic miR-6741-3p targets the oncogene SRSF3: Implications for oral squamous cell carcinoma pathogenesis
Source: PLoS One. 2024 May 23;19(5):e0296565. doi: 10.1371/journal.pone.0296565 (PMC11115324; doi:10.1371/journal.pone.0296565)
Supplement: S5 Fig — (PDF) [file pone.0296565.s005.pdf]

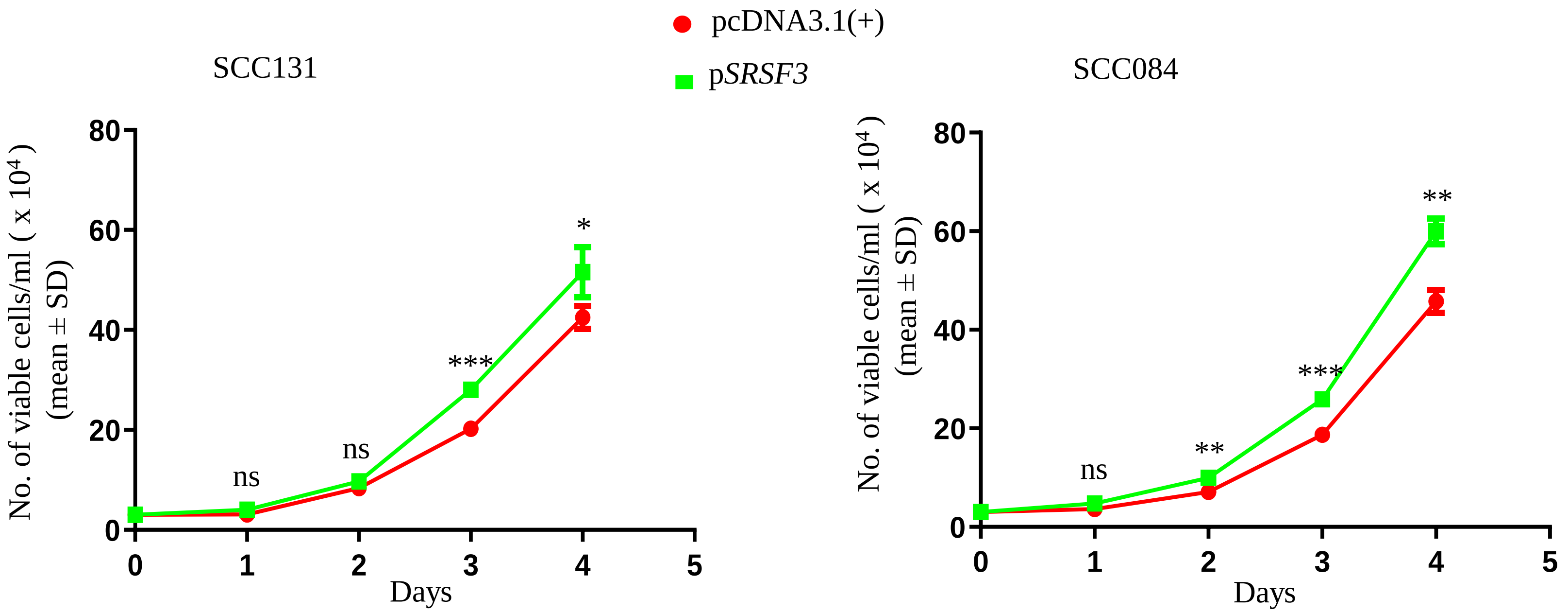

S5 Fig. SRSF3 overexpression increases the proliferation of SCC131 and SCC084 cells. The trypan blue dye exclusion assay revealed that the transient overexpression of *SRSF3* increases the proliferation of both SCC131 and SCC084 cells. Each data point is an average of 3 biological replicates.
