# Supplementary material for: Intronic miR-6741-3p targets the oncogene SRSF3: Implications for oral squamous cell carcinoma pathogenesis
Source: PLoS One. 2024 May 23;19(5):e0296565. doi: 10.1371/journal.pone.0296565 (PMC11115324; doi:10.1371/journal.pone.0296565)
Supplement: S6 Fig — (PDF) [file pone.0296565.s006.pdf]

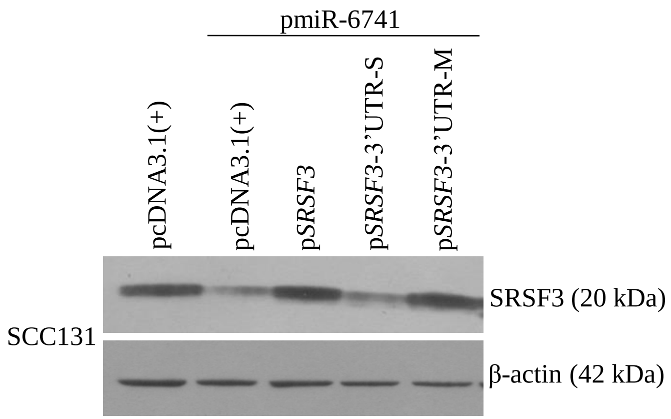

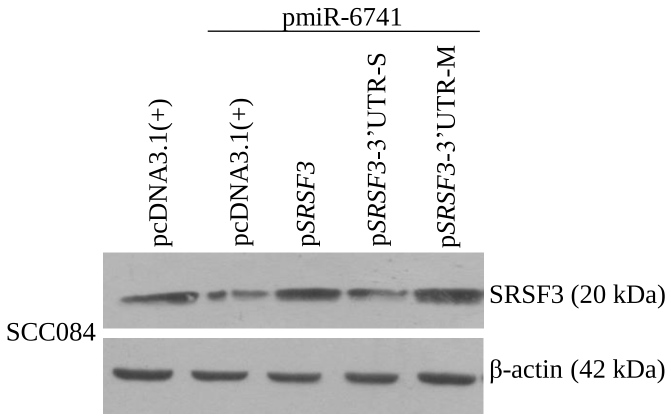

S6 Fig. SRSF3 expression depends on the presence or absence of its 3'UTR. The Western blot analysis of OSCC cells co-transfected with pmiR-6741 and different *SRSF3* constructs. Note, a reduced level of SRSF3 in SCC131 and SCC084 cells co-transfected with p*SRSF3*-3′UTR-S and pmiR-6741 in comparison to those co-transfected with pmiR-6741 and p*SRSF3* or pmiR-6741 and p*SRSF3*-3′UTR-M, underscoring that miR-6741-3p targets *SRSF3* by binding to its 3′UTR.
