# Supplementary material for: Intronic miR-6741-3p targets the oncogene SRSF3: Implications for oral squamous cell carcinoma pathogenesis
Source: PLoS One. 2024 May 23;19(5):e0296565. doi: 10.1371/journal.pone.0296565 (PMC11115324; doi:10.1371/journal.pone.0296565)
Supplement: S7 Fig — (PDF) [file pone.0296565.s007.pdf]

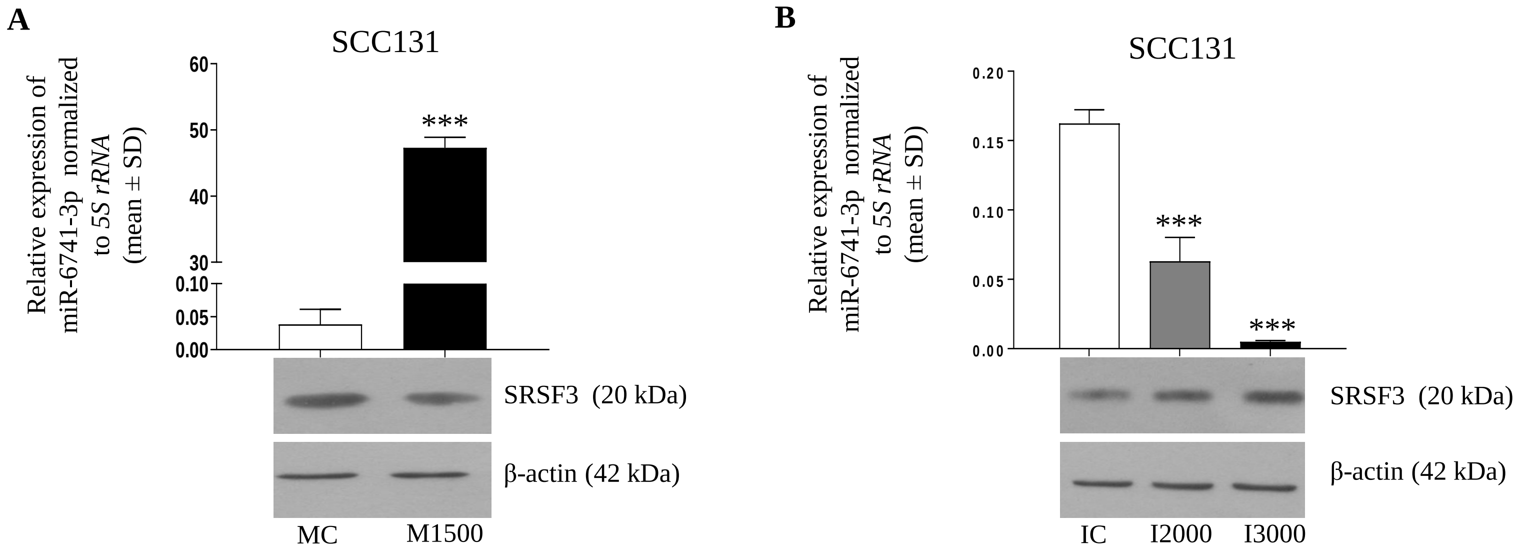

S7 Fig. Optimization of dosage for miR-6741-3p mimic and inhibitor in SCC131 cells. A) Cells were transfected with 1,500 nM of miR-6741-3p mimic (M1500) or 1500 nM mimic control (MC). B) Cells were transfected with 2,000 nM (I2000) and 3,000 nM (I3000) of miR-6741-3p inhibitor or 3,000 nM inhibitor control (IC), followed by Western blot analysis and qRT-PCR to check the levels of SRSF3 and miR-6741-3p respectively. Each qRT-PCR data is an average of 3 technical replicates.
