# Supplementary material for: Intronic miR-6741-3p targets the oncogene SRSF3: Implications for oral squamous cell carcinoma pathogenesis
Source: PLoS One. 2024 May 23;19(5):e0296565. doi: 10.1371/journal.pone.0296565 (PMC11115324; doi:10.1371/journal.pone.0296565)
Supplement: S8 Fig — (PDF) [file pone.0296565.s008.pdf]

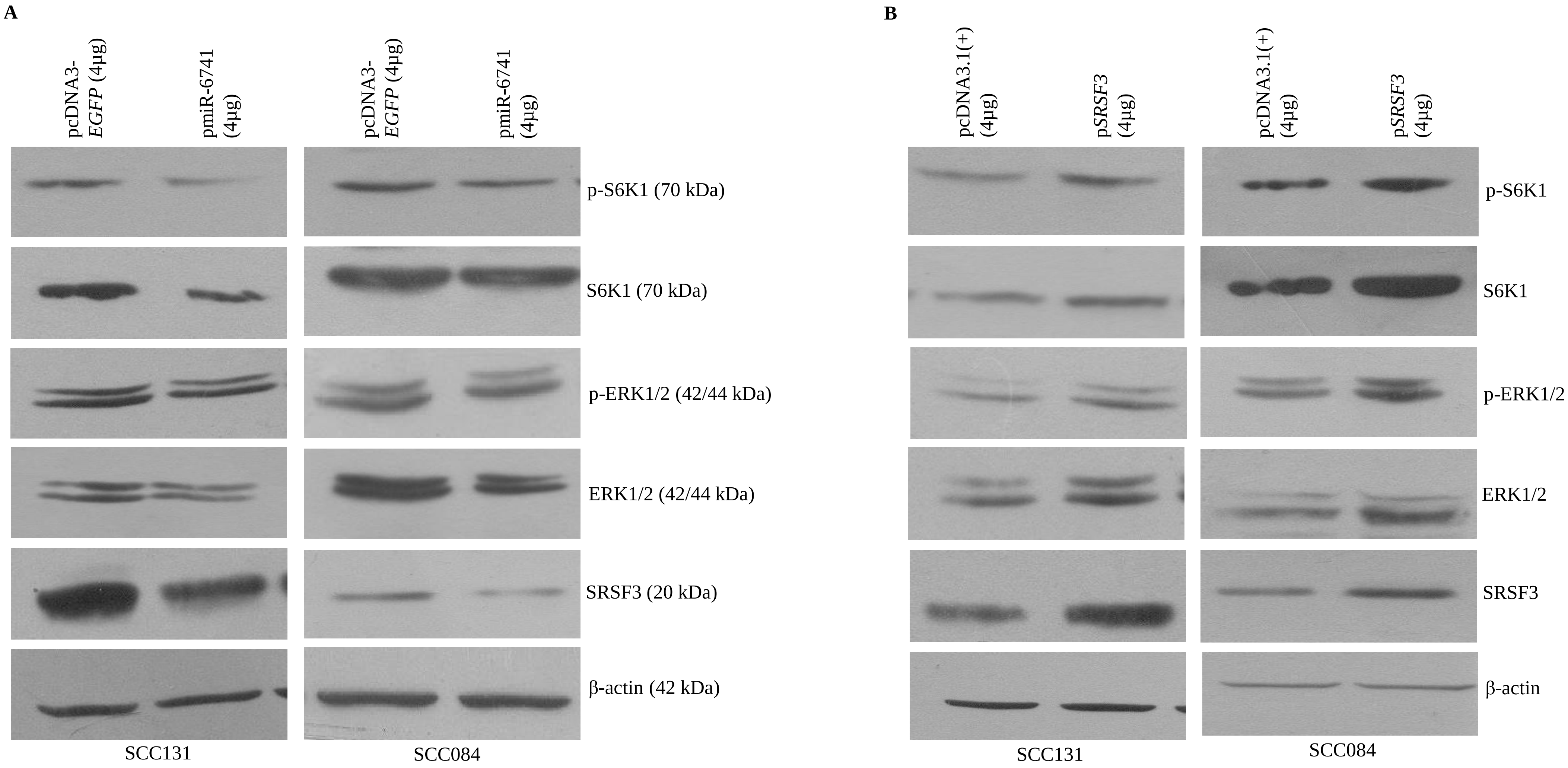

S8 Fig. MiR-6741-3p decreases signaling through both PI3K-AKT-MTOR and ERK/MAPK pathways, in part, by regulating SRSF3. A) Western blot analysis following overexpression of miR-6741-3p in SCC131 and SCC084 cells. B) Western blot analysis following overexpression of *SRSF3* in SCC131 and SCC084 cells.
