# Supplementary material for: Intronic miR-6741-3p targets the oncogene SRSF3: Implications for oral squamous cell carcinoma pathogenesis
Source: PLoS One. 2024 May 23;19(5):e0296565. doi: 10.1371/journal.pone.0296565 (PMC11115324; doi:10.1371/journal.pone.0296565)
Supplement: S9 Fig — (PDF) [file pone.0296565.s009.pdf]

A

5′CTCTTGCAGAAGATGGGTGTGAACCTGACACGCAGCAACAAGGAGACGGT
GAAGCACAGCGACGTCCTGTTTCTGGCTGTGAAGCCACATATCATCCCCT
TCATCCTGGATGAGATTGGGGCCGACGTGCAAGCCAGACACATCGTGGTC
TCCTGTGCGGCTGGTGTCACCATCAGCTCTGTGGAGAAGGTGCCCATCTC
AGCCCTGATGCCCCTTGTGTGAGGGGAGGGTTGACCCACGAATGGCAGCT
+1 +6 miR-6741-5p +27 +42 miR-6741-3p
AATGGG**TGGGTGC**TGGTGGGAGCCGTGCCCTGGCCACTCATT**CGGCTCT**C
+63
TCCCTCACCCTAGAAGCTGATGGCATTCCAGCCAGCCCCCAAAGTGATTC
GCTGCATGACCAACACACCTGTGGTAGTGCAGGAAGGCGCTACAGTGTAC
GCCACGGGCACCCATGCCCTGGTGGAGGATGGGCAGCTCCTGGAGCAGCT
CATGAGCAGCGTGGGCTTCTGCACTGAGGTGGAAGAGGACCTCATCGATG 3′

B

5′ AGTCGTGAATGAGCATATTGGGAAGAATCTGGTGAGTTAGTTGTTCTGTTCTCCAAAAACA
TATTCCAAGTCTTCACCCAAAACCTGTGCTCTGTGGGAGAGGATGGCTGATGAGGCAGGTG
ACAGGCAGTGTCTGTGCCTAGGGTGGGCTGATGTCCCTAGCACTGGAGGGTCCTGCTTCTC
AGTTTGGAGTCATAGAGGGATGGATGGCTTCCAGCCTAGCCCTGGACCACATGGAGACTGT
GCGGTCCCCACGAACAAGCGGCCAGGGTTAGGATAGGGCCTGACTCAGCTGTGCTTTTAAC
GACCAGGTATTAGCATTTCCCTTTGCCCTGCCACTTTCACCATAGGGCCTTCTTACCTGGC
AGAGGAGTGCCTTAGATACCAGAAGATTGGCAGGGAAGAAGGGCAGCCACTTCCTGGTTAC
CATGGAGAAGCTTGTCATGCTCCAAGCCTGTGCTTACTTGTCCAGTAGCAACAATGGGAAA
CTGTATTATTTGGGGTAGGGGTAGAACCCTGAGGGCATAAAGCTAAGAATTCCAGGCTGCA
TCTGGCAGAATCGGTTTGGCAGGGGTTCAGCTGCTCCCTGGGAGGCCTTGGCATAGCCAGG
CTGCTCCAGCACTGTGAGCTGGGAGTCTCCTCTTGCAGAAGATGGGTGTGAACCTGACACG
CAGCAACAAGGAGACGGTGAAGCACAGCGACGTCCTGTTTCTGGCTGTGAAGCCACATATC
ATCCCCTTCATCCTGGATGAGATTGGGGCCGACGTGCAAGCCAGACACATCGTGGTCTCCT
GTGCGGCTGGTGTCACCATCAGCTCTGTGGAGAAGGTGCCCATCTCAGCCCTGATGCCCCT
+1 +6 miR-6741-5p
TGTGTGAGGGGAGGGTTGACCCACGAATGGCAGCTAATGGG**TGGGTGC**TGGTGGGAGCCG
+27 +42 miR-6741-3p +63
TGCCCTGGCCACTCATT**CGGCTCT**CTCCCTCACCCTAGAAGCTGATGGCATTCCAGCCAG
CCCCCAAAGTGATTCGCTGCATGACCAACACACCTGTGGTAGTGCAGGAAGGCGCTACAGT
GTACGCCACGGGCACCCATGCCCTGGTGGAGGATGGGCAGCTCCTGGAGCAGCTCATGAGC
AGCGTGGGCTTCTGCACTGAGGTGGAAGAGGACCTCATCGATGCCGTCACGGGGCTCAGTG
GCAGCGGGCCTGCCTATGTGAGGCCCTCATTTGCTCGCTCAGCCTTCTAGGAACCCAGGCA
GCAAGATGGGCTGGCAGGCAGGCTTGGACTGGGGGTGGCGCCCACCTCTTGGGCACAGCTG
GCTGGTACAGGATGCTGACCCTTGGAGCTAGTTCTAGTCATCAGAAAGCAGACCTAAGGAA
AGGCCCTTCAGTGCCTATGGCCTGGGGCTTAGTGAGTGTCTCCACAGGCATTCATGGCTCT
GGACGCATTGGCTGATGGTGGGGTGAAGATGGGTTTGCCACGGCGCCTGGCAATCCAACTC
GGGGCCCAGGCTTTGCTGGTCAGTATCTTTCCCCCGCATGTTCAGGACCAGGGTGTAGAAT
GGGGGTTCTTGTGCATCACTGAACTGAGGTGGGAGTTGGTTGGGAAGCTGGGGTAGCAGTT
GGGCAG GAGCAGTGGGTATGGGAAGCCTGTTC 3′

C

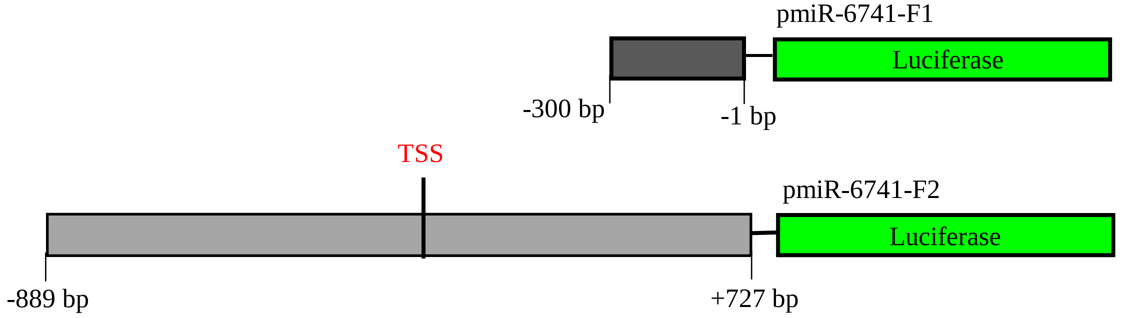

S9 Fig. Putative promoter sequence for the *MIR6741* gene. A) The promoter region of *MIR6741* predicted by the DBTSS database. B) The promoter region of *MIR6741* predicted by the DBTSS database along with additional upstream and downstream sequences retrieved from the UCSC Genome Browser. TSS represents the transcription start site and is numbered as +1, and the rest of the sequence is numbered relative to it. The pre-miRNA sequence of miR-6741 is colored red and underlined. The mature miR-6741-5p and miR-6741-3p sequences are highlighted in yellow and cyan respectively. C) Schematic diagrams of the two putative *MIR6741* promoter constructs. *Abbreviations:* TSS, Transcription start site; and, bp, base pair.
