# Supplementary material for: Intronic miR-6741-3p targets the oncogene SRSF3: Implications for oral squamous cell carcinoma pathogenesis
Source: PLoS One. 2024 May 23;19(5):e0296565. doi: 10.1371/journal.pone.0296565 (PMC11115324; doi:10.1371/journal.pone.0296565)
Supplement: S10 Fig — (PDF) [file pone.0296565.s010.pdf]

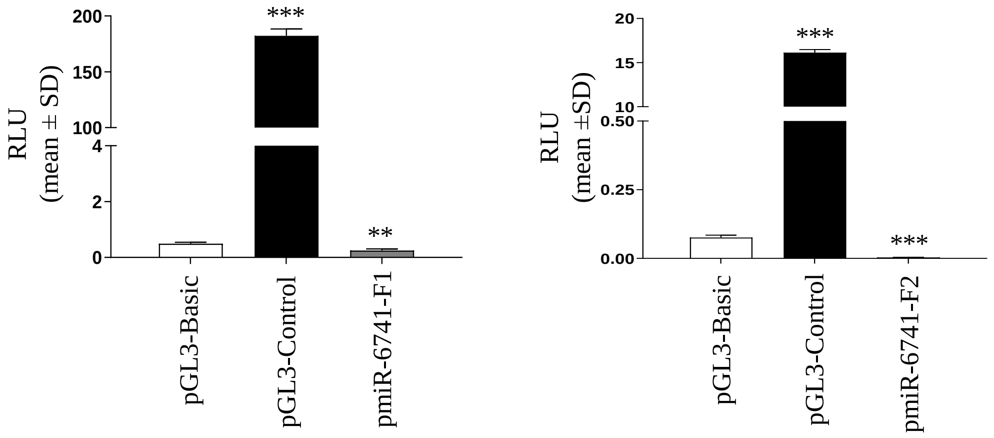

S10 Fig. The dual-luciferase reporter assay for putative *MIR6741* promoter fragments in SCC131 cells. Note, no promoter activity was observed for the *MIR6741* promoter constructs. pGL3-Control harbours SV40 promoter and was used as a positive control. Each bar is an average of 3 biological replicates.
