# Supplementary material for: Intronic miR-6741-3p targets the oncogene SRSF3: Implications for oral squamous cell carcinoma pathogenesis
Source: PLoS One. 2024 May 23;19(5):e0296565. doi: 10.1371/journal.pone.0296565 (PMC11115324; doi:10.1371/journal.pone.0296565)
Supplement: S11 Fig — (PDF) [file pone.0296565.s011.pdf]

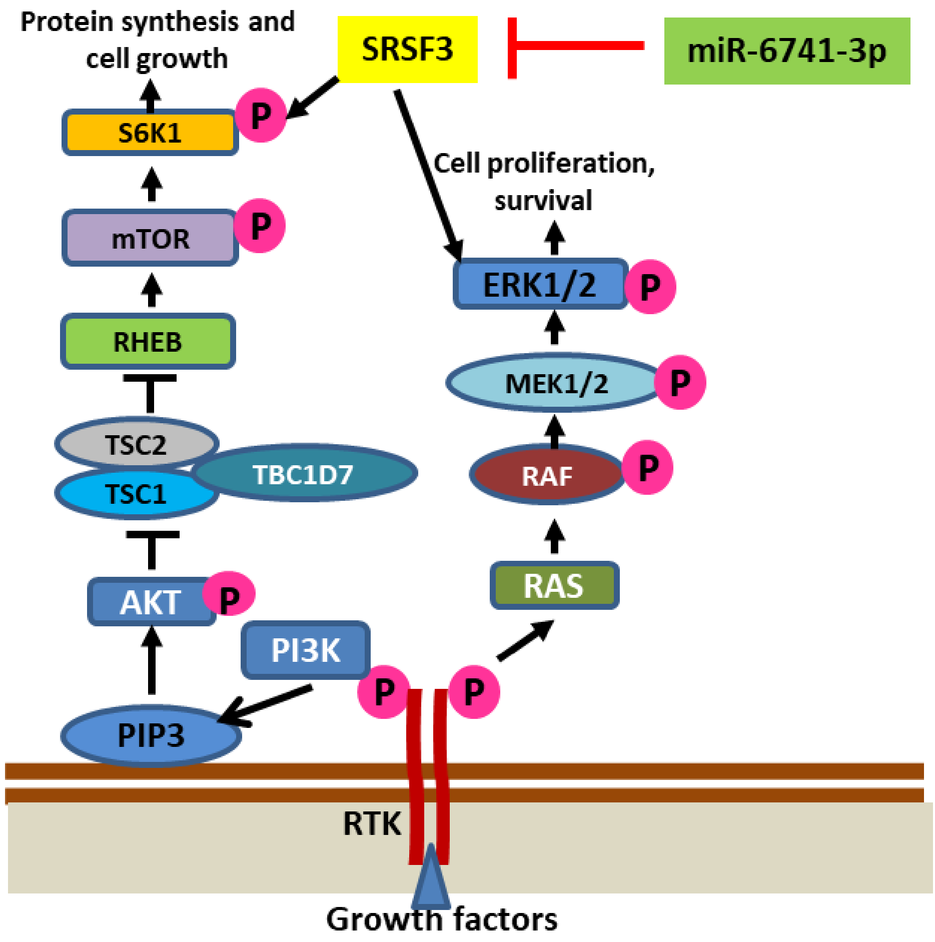

S11 Fig. Effect of miR-6741-3p-mediated regulation of SRSF3 on the PI3K/AKT/MTOR and the ERK/MAPK pathways. Mature miR-6741-3p targets the target site located in the 3'UTR of *SRSF3* mRNA and represses its translation, leading to a decreased level of SRSF3 protein. The decreased SRSF3 level in turn decreases the signaling through both the PI3K/AKT/MTOR and the ERK/MAPK pathways. The figure was drawn using Microsoft PowerPoint version 2010. *Abbreviation:* RTK, Receptor tyrosine kinase.
