# Supplementary material for: Intronic miR-6741-3p targets the oncogene SRSF3: Implications for oral squamous cell carcinoma pathogenesis
Source: PLoS One. 2024 May 23;19(5):e0296565. doi: 10.1371/journal.pone.0296565 (PMC11115324; doi:10.1371/journal.pone.0296565)
Supplement: S1 Table — (PDF) [file pone.0296565.s012.pdf]

S1 Table. Differentially expressed microRNAs identified by microRNA microarray analysis of 5-Azacytidine and DMSO-treated SCC131 cells.

List of upregulated microRNAs

Sl. No	Name of microRNA	Mean fold expression	P-Value
1	miR-5008-5p	4.16	0.0000
2	miR-198	4.11	0.0000
3	miR-664a-3p	5.26	0.0000
4	miR-5096	4.00	0.0000
5	miR-516b-5p	4.67	0.0000
6	miR-654-5p	1.56	0.0000
7	miR-6510-5p	0.84	0.0000
8	miR-6732-3p	4.48	0.0000
9	miR-4317	4.27	0.0000
10	miR-550b-2-5p	1.52	0.0000
11	miR-4687-5p	4.28	0.0001
12	miR-3940-3p	5.82	0.0001
13	miR-885-5p	6.21	0.0001
14	miR-631	4.39	0.0001
15	miR-500a-3p	0.89	0.0001
16	miR-4758-3p	4.81	0.0002
17	miR-4763-5p	5.26	0.0003
18	miR-6741-3p	4.96	0.0003
19	miR-1299	4.31	0.0006
20	miR-7152-5p	4.93	0.0007
21	miR-3622a-5p	1.06	0.0007
22	miR-520e	4.27	0.0010
23	miR-187-5p	3.63	0.0019
24	miR-4652-3p	4.16	0.0024
25	miR-6756-3p	1.71	0.0054
26	miR-7114-3p	0.93	0.0058
27	miR-4728-3p	0.89	0.0063
28	miR-6779-3p	1.74	0.0066
29	miR-6743-3p	1.16	0.0070
28	miR-6779-3p	1.74	0.0066

29	miR-6743-3p	1.16	0.0070
30	miR-6858-3p	0.90	0.0098
31	miR-4649-3p	0.88	0.0129
32	miR-744-3p	5.59	0.0162
33	miR-197-3p	0.97	0.0165
34	miR-6855-3p	1.55	0.0181
35	miR-6804-3p	1.12	0.0230
36	miR-3935	0.94	0.0250
37	miR-5585-3p	1.20	0.0314
38	miR-520b	1.16	0.0347
39	miR-6865-5p	1.06	0.0355
40	miR-483-3p	0.90	0.0358
41	miR-3131	5.16	0.0395
42	miR-3620-3p	1.15	0.0488
43	miR-6815-3p	4.83	0.0716
44	miR-1229-3p	4.24	0.0722
45	miR-6892-5p	4.65	0.0735
46	miR-4668-5p	4.29	0.0940
47	miR-7109-5p	4.23	0.1159
48	miR-617	3.50	0.1182
49	miR-6731-3p	1.24	0.1499
50	miR-7113-5p	1.00	0.1733

List of downregulated

Sl. No	Name of the microRNA	Mean fold expression	P-Value
1	miR-4697-5p	-5.41	0.0000
2	miR-5684	-5.57	0.0000
3	miR-665	-3.55	0.0000
4	miR-494-3p	-2.01	0.0000
5	miR-4446-3p	-4.51	0.0000
6	miR-6511b-5p	-1.73	0.0000
7	miR-4685-5p	-1.43	0.0000
8	miR-1273g-3p	-1.40	0.0000
9	miR-4298	-0.85	0.0000
10	miR-6515-3p	-0.86	0.0000
11	miR-501-5p	-4.10	0.0000
12	miR-513c-5p	-1.26	0.0000
13	miR-3616-3p	-5.23	0.0000
14	miR-5100	-1.00	0.0001
15	miR-431-3p	-0.86	0.0003
16	miR-671-3p	-0.83	0.0007
17	miR-3124-5p	-0.90	0.0009
18	miR-4513	-1.31	0.0050
19	miR-6805-5p	-1.28	0.0059
20	miR-6817-5p	-1.05	0.0109
21	miR-664b-5p	-0.91	0.0201
22	miR-6795-5p	-4.70	0.0240
23	miR-6868-5p	-3.95	0.0443
24	miR-1304-3p	-3.90	0.0450

25	miR-191-3p	-3.79	0.0464
26	miR-4731-3p	-2.92	0.0939
27	miR-6795-3p	-2.62	0.1222
28	miR-6889-3p	-2.60	0.1578
