# Supplementary material for: Intronic miR-6741-3p targets the oncogene SRSF3: Implications for oral squamous cell carcinoma pathogenesis
Source: PLoS One. 2024 May 23;19(5):e0296565. doi: 10.1371/journal.pone.0296565 (PMC11115324; doi:10.1371/journal.pone.0296565)
Supplement: S2 Table — (PDF) [file pone.0296565.s013.pdf]

S2 Table. Details of primers used in qRT-PCR.

Gene	Sequence (5' to 3')	Amplicon size (bp)	Annealing temp. (ºC)	Reference
GAPDH	F: GAAGGGTGAAGGTCGGAGTC<br>R: GAAGATGGTGATGGGATTTC	226	60	–
MIR6741 (miR-6741-3p)	RT6-miR-6741-3p:<br>TGTCAGGCAACCGTATTCACCGTGAGTGGTCTAGGG<br>Short-miR-6741-3p:<br>CGTCAGATGTCCGAGTAGAGGGGGAACGGCGTCGGCT CTCTCCCTCACCC<br>MP- fw: TGTCAGGCAACCGTATTCACC<br>MP- rev: CGTCAGATGTCCGAGTAGAGG	86	54	[49], Present study
5S rRNA	F: GCCCGATCTCGTCTGATCT<br>R: AGCCTACAGCACCCGGTATT	93	60	–
MCPH1	F: TCACCACAGCGCAATGGAGAAGAGA<br>R: ATCACGTGAAATGTTCAAAGGTGCTTC	109	62	[16]
SRSF3	F: ATGGAAGAACACTATGTGGCTGCCGTG<br>R: TCTCTAGAAAGGGACCTGCTCCGGCT	196	56	–

Abbreviations: F, forward primer; R, reverse primer; fw, forward; rev, reverse; bp, base pair; and, temp., temperature.
