# Supplementary material for: Intronic miR-6741-3p targets the oncogene SRSF3: Implications for oral squamous cell carcinoma pathogenesis
Source: PLoS One. 2024 May 23;19(5):e0296565. doi: 10.1371/journal.pone.0296565 (PMC11115324; doi:10.1371/journal.pone.0296565)
Supplement: S3 Table — (PDF) [file pone.0296565.s014.pdf]

S3 Table. A list of predicted gene targets^ for miR-6741-3p.

miRNA prediction programs		
**miRDB**	**DIANA-microT-CDS**	**TargetScan**
MAB21L1	*RBM19*	*ADM5*
MKX	*POLR1C*	*ASB6*
UBQLN1	*SRSF4*	*GPR1*
KCNB1	*SCAP*	*SRSF4*
FOXF1	***MKX***	*MAB21L1*
NLK	***SRSF3***	***SRSF3***
NDST2	*ATRNL1*	*SELM1*
SRSF3	***C6ORF89***	*FOXF1*
WDR78	***NDST2***	*SCAP*
HOTS	*UBQLN1*	*HIC2*
STK40	*PER2*	***MKX***
RABIF	*KCNB1*	***C6ORF89***
C6ORF89	*CDKL1*	*ATRNL1*
VPS33B	*PIGT*	***NDST2***
LBR	*RBFOX2*	*PER2*

^Gene targets highlighted in bold were predicted by all the three target prediction programs, of which *SRSF3* was further investigated in the present study.
