# Supplementary material for: Intronic miR-6741-3p targets the oncogene SRSF3: Implications for oral squamous cell carcinoma pathogenesis
Source: PLoS One. 2024 May 23;19(5):e0296565. doi: 10.1371/journal.pone.0296565 (PMC11115324; doi:10.1371/journal.pone.0296565)
Supplement: S4 Table — (PDF) [file pone.0296565.s015.pdf]

S4 Table. Clinicopathological parameters of the patients included in the study.

Sl. No.	Pt. No.	Age (yr)	Sex	Site of cancer	Tumor grade	TNM	Differentiation	Habit
1	2	65	F	BM	II	T4aN2M0	Moderate	Nil
2	3	56	F	BM	II	T3N2M1	Well	Betel nut + Tobacco chewing
3	5	48	M	BM + GBS	III	T4aN2M1	Well	Tobacco chewing
4	6	76	F	Alveolus	I	T4aN1M0	Well	Tobacco chewing
5	8	53	M	Tongue	I	T3aN1M1	Well	Pan + Tobacco chewing
6	10	31	M	BM	I	T4aN1M0	Well	Tobacco chewing
7	14	60	F	BM	I	T4aN1M0	Well	Tobacco chewing
8	17	58	F	BM	II	T4aN2M1	Well	Betel nut chewing
9	31	62	F	BM	II	T4N2M0	Well	Tobacco chewing
10	32	60	F	BM + GBS	I	T4aN1M0	Well	Tobacco chewing
11	33	45	F	GBS	I	T3aN1M0	Moderate	Betel nut chewing
12	43	39	F	RMT	I	T4N1M0	Well	Betel nut + Tobacco chewing
13	44	58	F	RMT	I	T4aN1M0	Well	Betel nut chewing
14	45	65	F	GBS + Maxilla	II	T4N2M0	Moderate	Betel nut + Tobacco chewing
15	46	39	M	GBS + RMT	II	T3aN2M0	Poor	Tobacco chewing
16	47	54	M	BM + GBS + Maxilla	I	T3aN1M0	Poor	Tobacco chewing
17	48	65	M	GBS + RMT	II	T4N2M0	Well	Pan Masala
18	49	78	F	GBS	II	T3N2M0	Moderate	Betel nut chewing
19	50	33	M	Tongue	II	T4N2M0	Well	Pan Masala
20	51	41	F	BM	I	T4aN1M0	Moderate	Betel nut + Tobacco chewing
21	52	73	F	BM	I	T3aN1M0	Moderate	Betel nut chewing
22	53	65	F	BM + GBS	I	T3aN1M0	Moderate	Betel nut + Tobacco chewing
23	54	65	F	BM + GBS	II	T2N2M1	Poor	Tobacco chewing
24	55	70	F	BM + GBS	I	T3aN1M0	Well	Nil
25	56	69	F	GBS	II	T3aN2M0	Moderate	Betel nut + Tobacco chewing
26	57	72	M	Alveolus	I	T4aN1M0	Well	Tobacco chewing
27	59	31	M	GBS + RMT	I	T4aN1M0	Well	Tobacco chewing
28	60	49	F	BM + GBS	III	T4aN2M1	Moderate	Betel nut chewing
29	61	60	M	BM	I	T4aN2M0	Poor	Betel nut + Tobacco chewing
30	62	53	M	Tongue	I	T3aN1M0	Moderate	Nil
31	63	52	F	BM	I	T2N1M0	Moderate	Betel nut chewing
32	64	45	M	BM + GBS + RMT	II	T3aN2M0	Well	Pan
33	65	44	M	Tongue	I	T4aN1M0	Well	Pan + Tobacco chewing
34	66	40	M	BM	II	T4aN2M0	Well	Tobacco chewing
35	67	43	F	BM + GBS	II	T4aN2M0	Well	Pan
36	68	28	M	Tongue	I	T2N1M0	Moderate	Gutkha + Tobacco chewing + Cigarette

Abbreviations: Pt. No., patient number; yr, years; M, male; F, female; TNM, Tumor Node and Metastasis; BM, buccal mucosa; GBS, gingivo-buccal sulcus; and, RMT, retromolar trigone.
