# Supplementary material for: Intronic miR-6741-3p targets the oncogene SRSF3: Implications for oral squamous cell carcinoma pathogenesis
Source: PLoS One. 2024 May 23;19(5):e0296565. doi: 10.1371/journal.pone.0296565 (PMC11115324; doi:10.1371/journal.pone.0296565)
Supplement: S5 Table — (PDF) [file pone.0296565.s016.pdf]

S5 Table. Details of plasmid constructs used in the study.

Construct	Cloning vector/ construct	Primer sequence (5' to 3')	Amplicon size (bp)	Annealing temp. (°C)
pmiR-6741	pcDNA3-*EGFP*	F: TAGCAAGCTTGGATGAGATTGGGGCCGACGTGC<br>*Hind* III<br>R: AGCTCTCGAGGGCATGGGTGCCCGTGGCGTAC<br>*Xho* I	311	56
p*SRSF3*	pcDNA3.1(+)	F: GTCTACGGTACCTTAACCCTAGATCTCGAAATGCATCGT<br>*Kpn* I<br>R: ACTGCAGGATCCCTATTTCCTTTCATTTGACCTAGATCGA<br>*Bam* HI	513	62
pMIR-REPORT-*SRSF3*-3'UTR-S	pMIR-REPORT™	F: GTAACGCGTATCGGATCCAAGACAGTTTGCAAGAGAAGTGGTGTAC<br>*Mlu* I *Bam* HI<br>R: CATGTTTAAACTATGATATCTGTGAATCTTGCCATGTTGGGCAGACT<br>*Pme* I *Eco* RV	2450	72
pmiR-6741-F1	pGL3-Basic	F: GACTGCTCGAGGGAGGCCTTGGCATAGCCAG<br>*Xho* I<br>R: GACTGAAGCTTAGCTGCCATTCGTGGGTCAAC<br>*Hind* III	300	56
pmiR-6741-F2	pGL3-Basic	F: GACTGACGCGTAGTCGTGAATGAGCATATTGGGAAGAATC<br>*Mlu* I<br>R: GACTGCTCGAGGAACAGGCTTCCCATACCCACTGCTC<br>*Xho* I	1616	72
**Plasmid constructs generated by sub-cloning**				
p*SRSF3*-3'UTR-S	p*SRSF3*	F: GTAACGCGTATCGGATCCAAGACAGTTTGCAAGAGAAGTGGTGTAC<br>*Mlu* I *Bam* HI*<br>R: CATGTTTAAACTATGATATCTGTGAATCTTGCCATGTTGGGCAGACT<br>*Pme* I *Eco* RV*	–	–
p*SRSF3*-3'UTR-M	p*SRSF3*	F: GTAACGCGTATCGGATCCAAGACAGTTTGCAAGAGAAGTGGTGTAC<br>*Mlu* I *Bam* HI*<br>R: CATGTTTAAACTATGATATCTGTGAATCTTGCCATGTTGGGCAGACT<br>*Pme* I *Eco* RV*	–	–

Abbreviations: F, forward primer; R, reverse primer; bp, base pair; and, temp., temperature. * Restriction enzymes used for sub-cloning in the p*SRSF3* construct.
