# Supplementary material for: Intronic miR-6741-3p targets the oncogene SRSF3: Implications for oral squamous cell carcinoma pathogenesis
Source: PLoS One. 2024 May 23;19(5):e0296565. doi: 10.1371/journal.pone.0296565 (PMC11115324; doi:10.1371/journal.pone.0296565)
Supplement: S6 Table — (PDF) [file pone.0296565.s017.pdf]

S6 Table. Details of construct generated by site-directed mutagenesis in the study.

Construct	Template vector	Primer sequence (5' to 3')
pMIR-REPORT-*SRSF3*-3'UTR-M	pMIR-REPORT-*SRSF3* 3'UTR-S	F: AAGGGTACATTGTATCTAAGAACTTTGAGTTACT<br>R: AGTAACTCAAAGTTCTTAGATACAATGTACCCTT

Abbreviations: F, forward primer; and, R, reverse primer.
