# Supplementary material for: Intronic miR-6741-3p targets the oncogene SRSF3: Implications for oral squamous cell carcinoma pathogenesis
Source: PLoS One. 2024 May 23;19(5):e0296565. doi: 10.1371/journal.pone.0296565 (PMC11115324; doi:10.1371/journal.pone.0296565)
Supplement: S7 Table — (PDF) [file pone.0296565.s018.pdf]

S7 Table. Details of the putative *MIR6741* promoter fragments cloned in the pGL3-Basic vector.

Construct	Database used for promoter prediction	Promoter fragment position relative to TSS	Fragment length (bp)
pmiR-6741-F1	DBTSS	-300 to -1	300
pmiR-6741-F2	DBTSS*	-889 to +727	1616

Abbreviations: TSS, Transcription Start Site; and, bp, base pair. *Additional sequence upstream and downstream of the sequence predicted by DBTSS was included.
