# Supplementary material for: Intronic miR-6741-3p targets the oncogene SRSF3: Implications for oral squamous cell carcinoma pathogenesis
Source: PLoS One. 2024 May 23;19(5):e0296565. doi: 10.1371/journal.pone.0296565 (PMC11115324; doi:10.1371/journal.pone.0296565)
Supplement: S1 Raw images — (PDF) [file pone.0296565.s019.pdf]

A

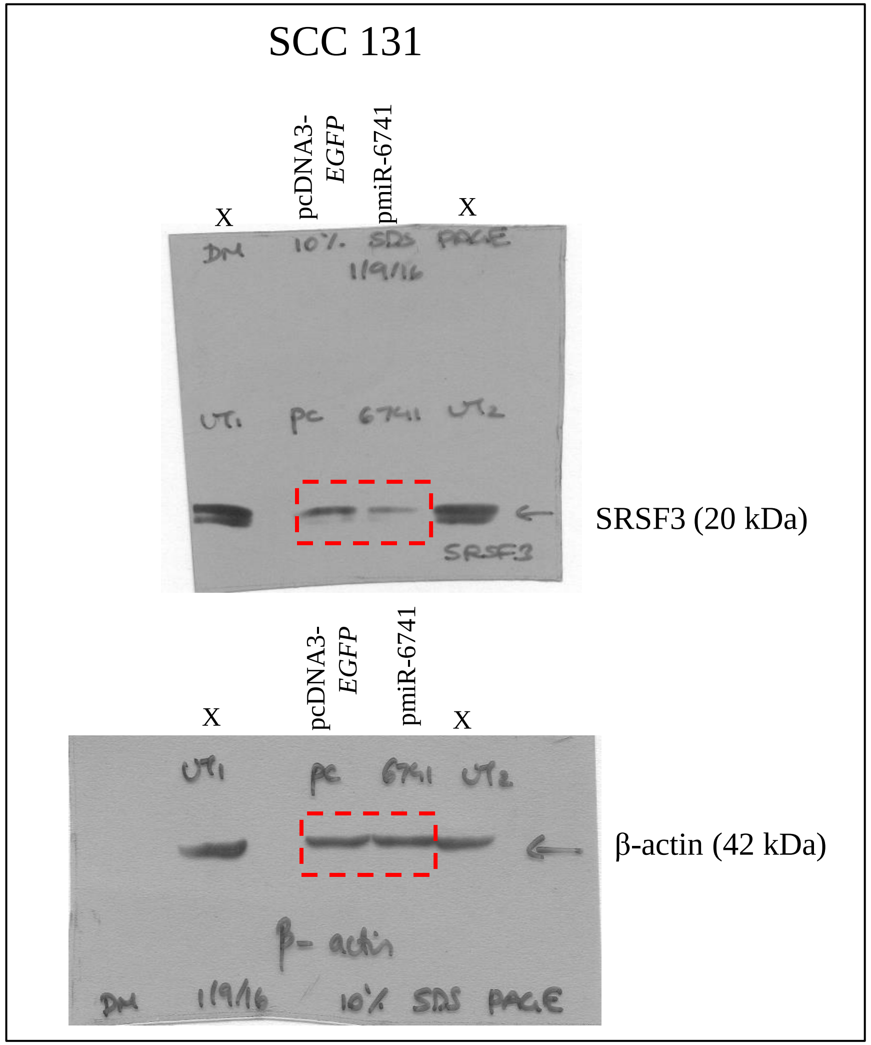

B

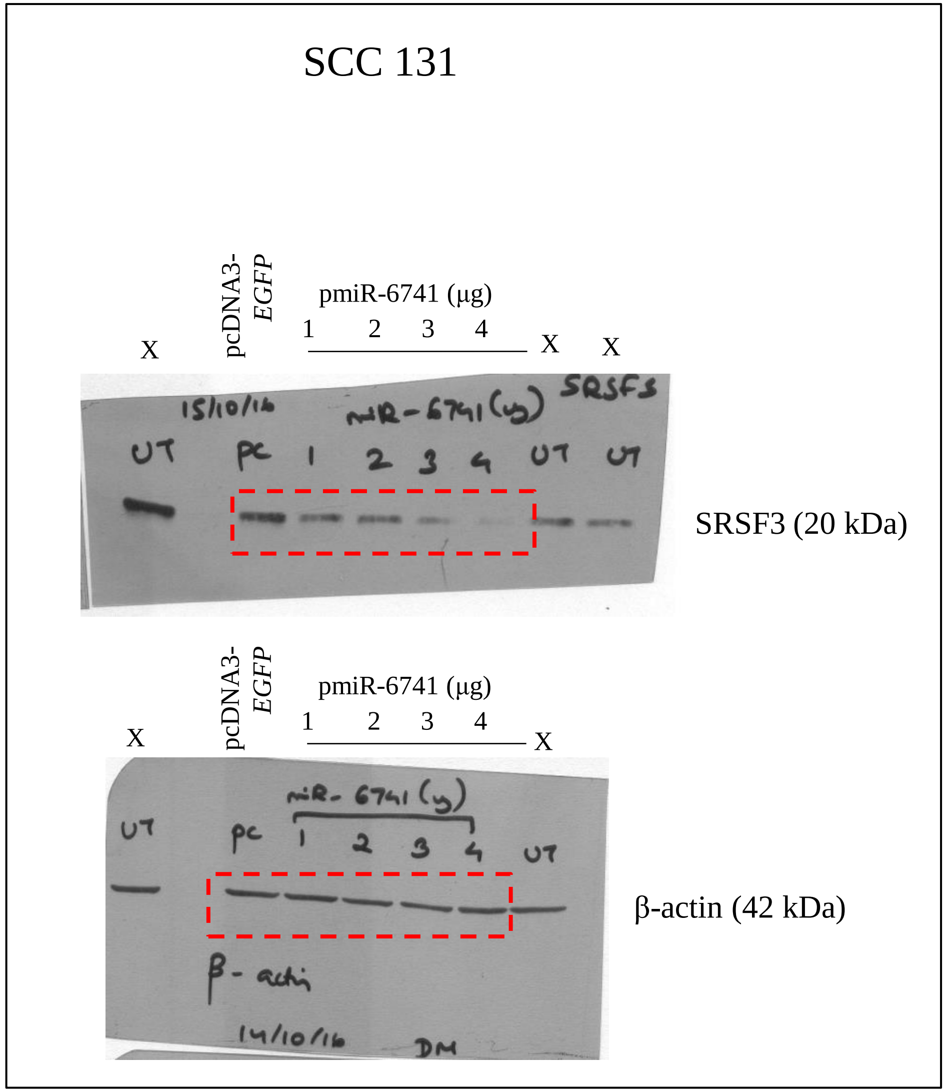

A) Full-length blots of Fig 2A. B) Full-length blots of Fig 2B.

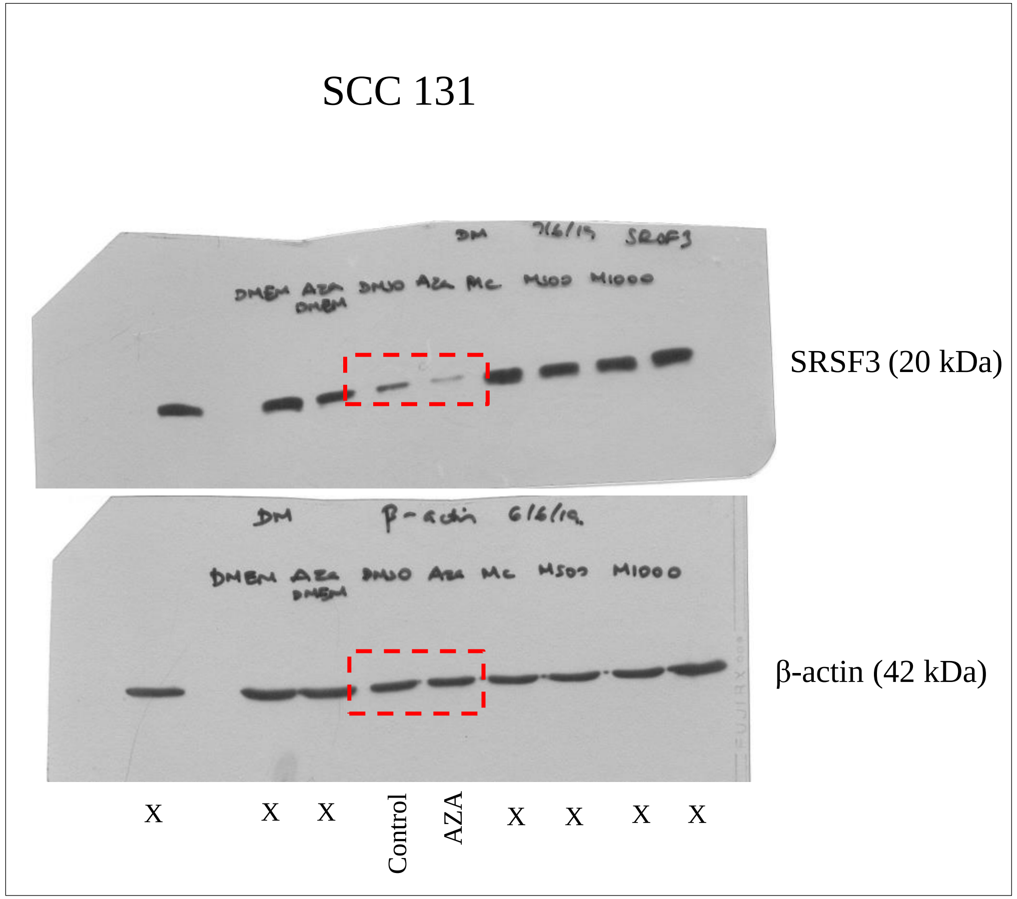

Full-length blots for S2 Fig.

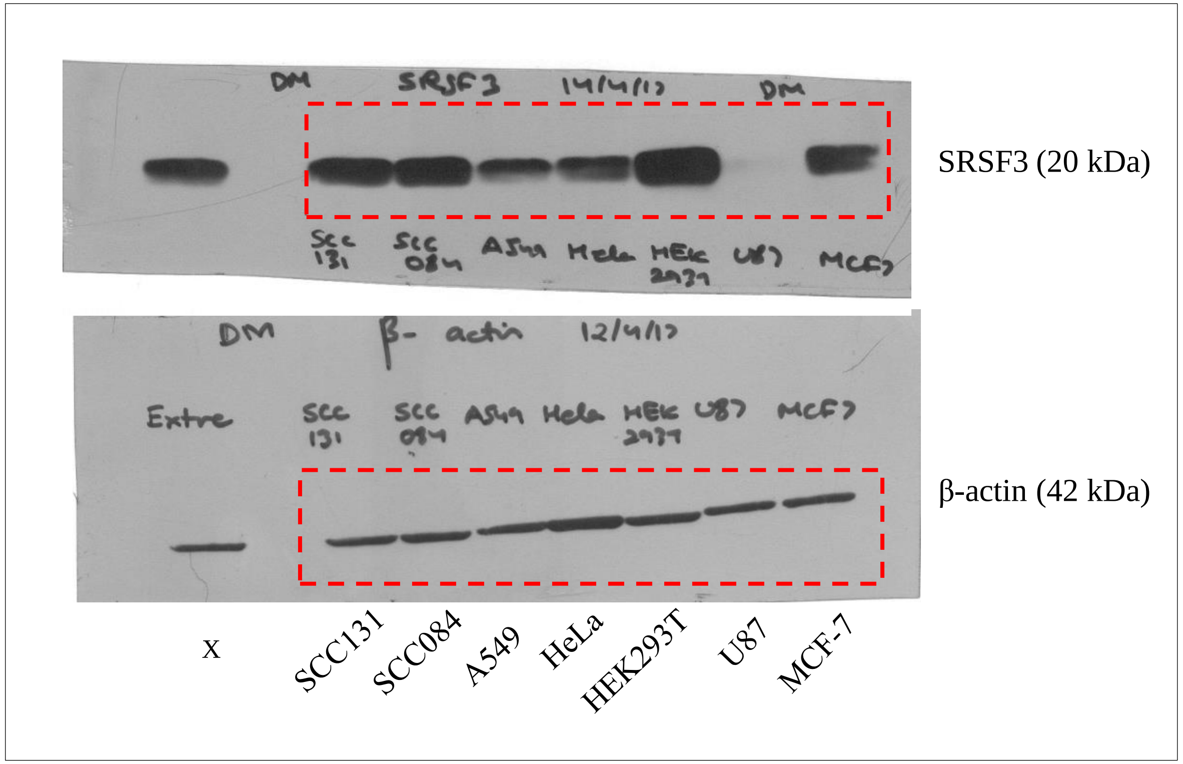

Full-length blots for S3 Fig.

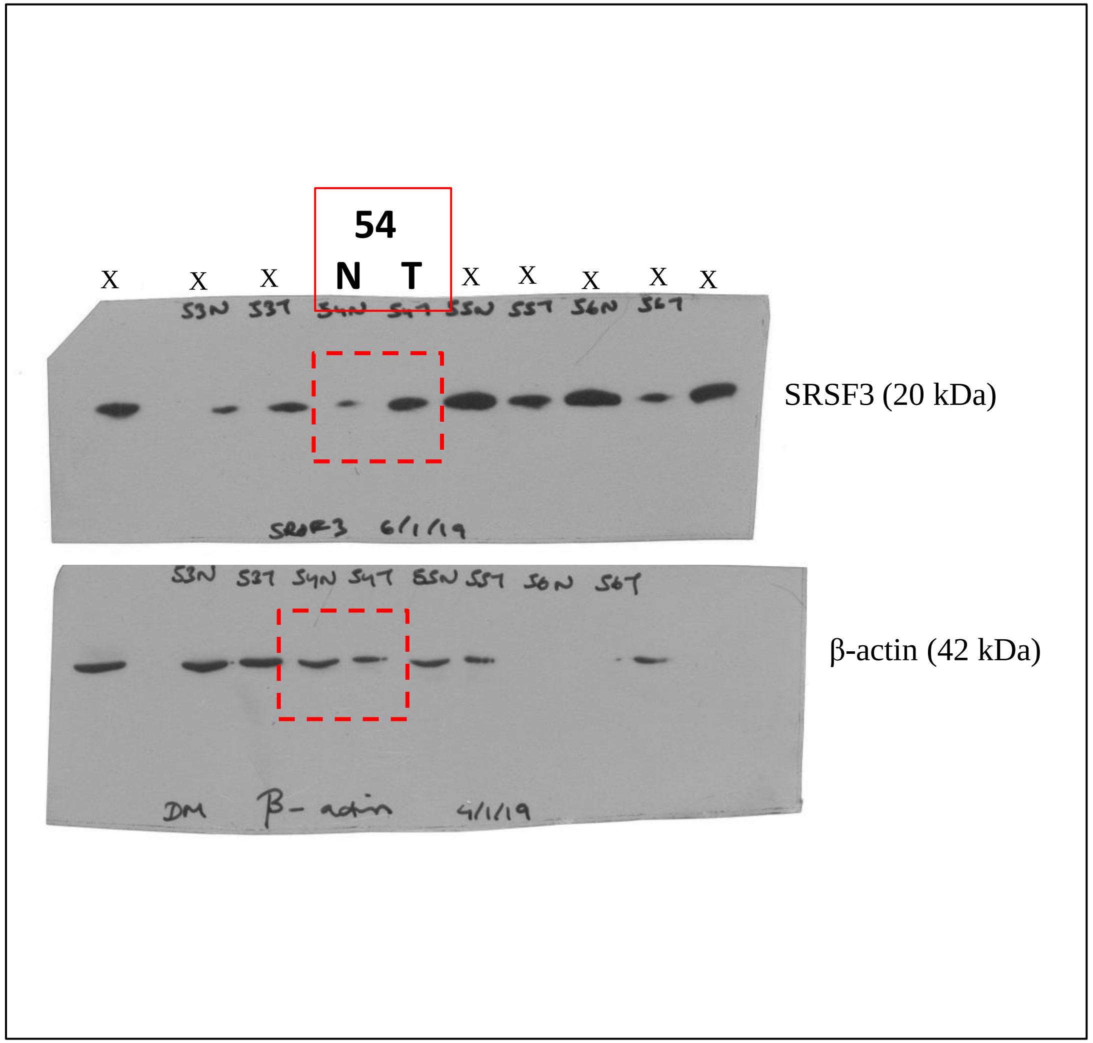

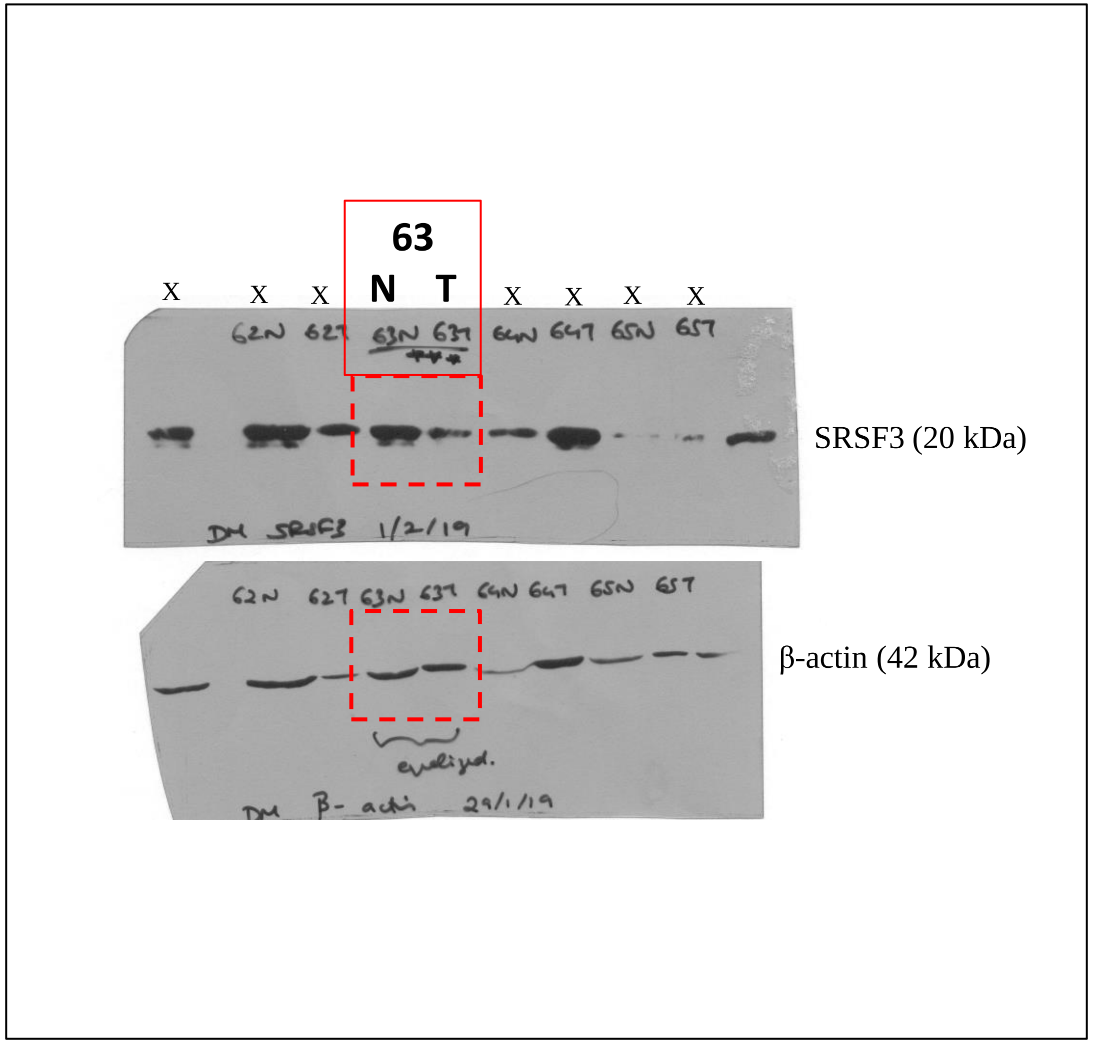

Full-length blots for S4 Fig.

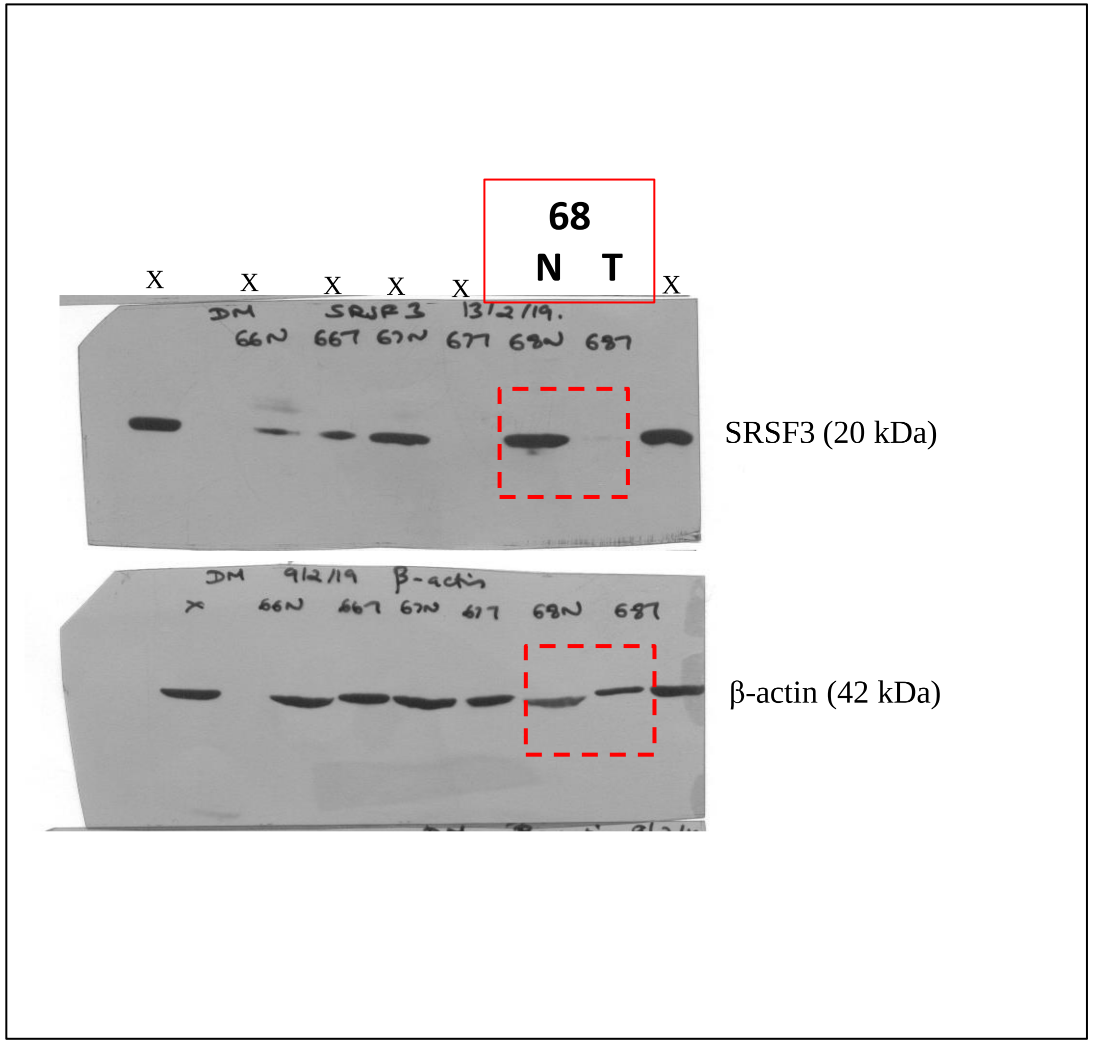

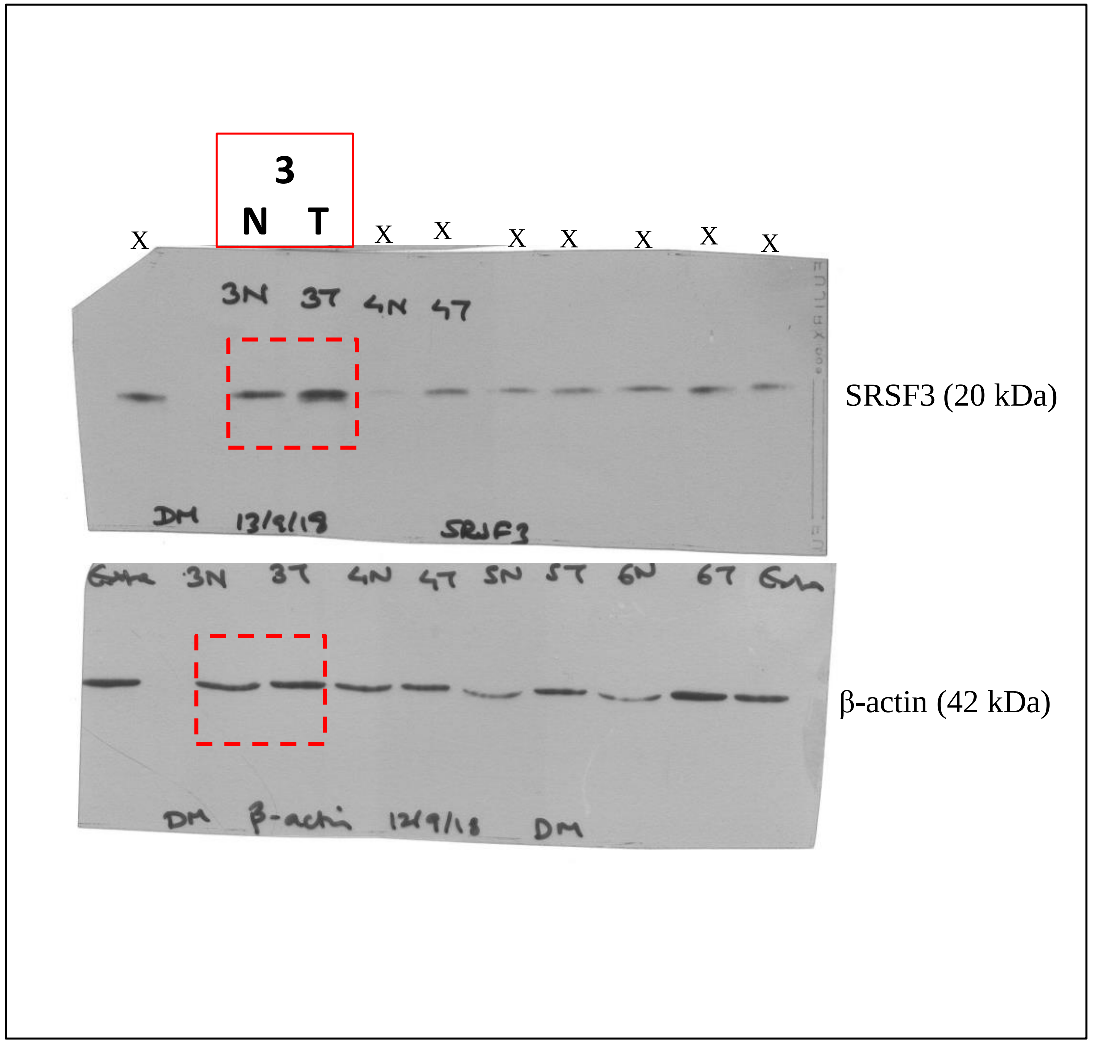

Full-length blots for S4 Fig.

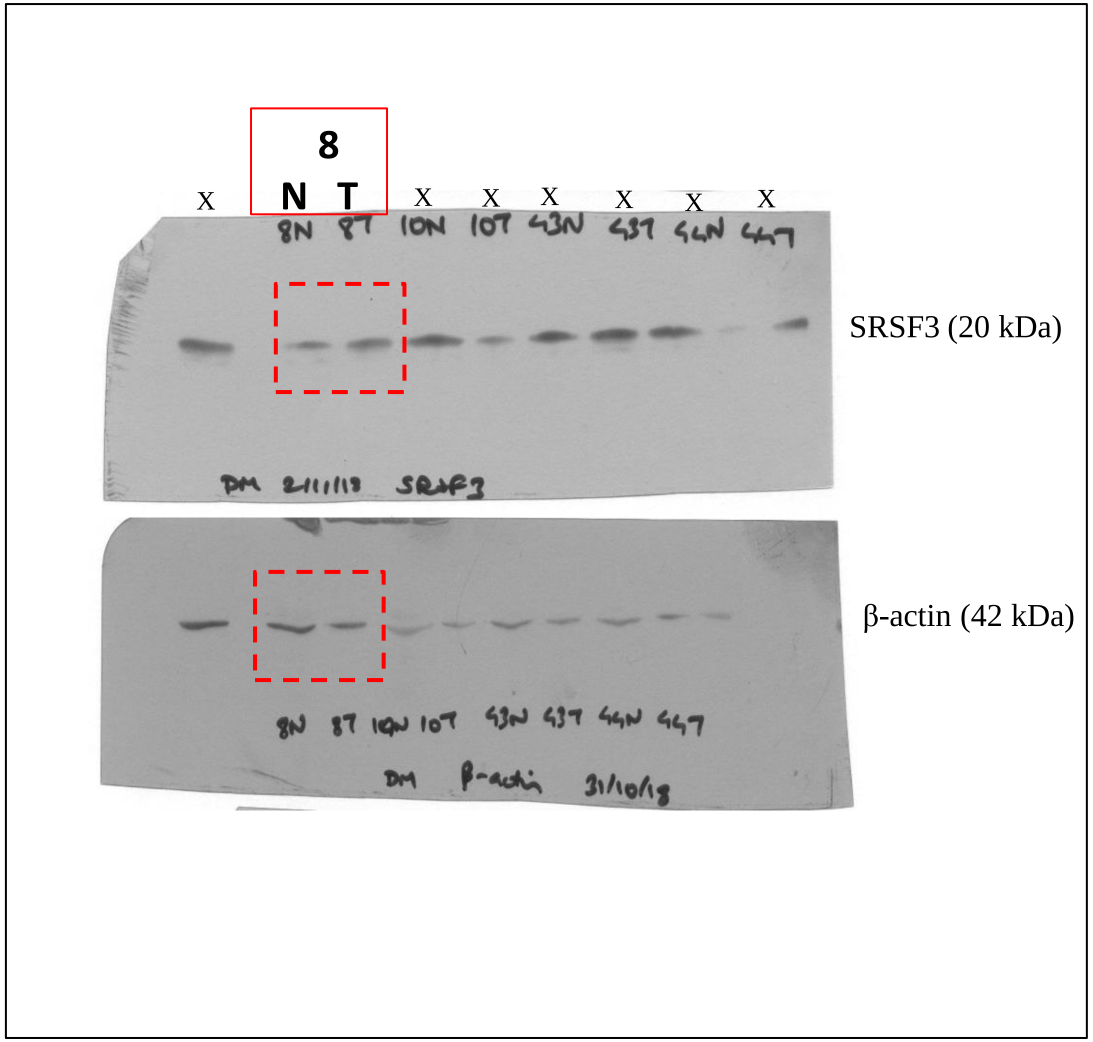

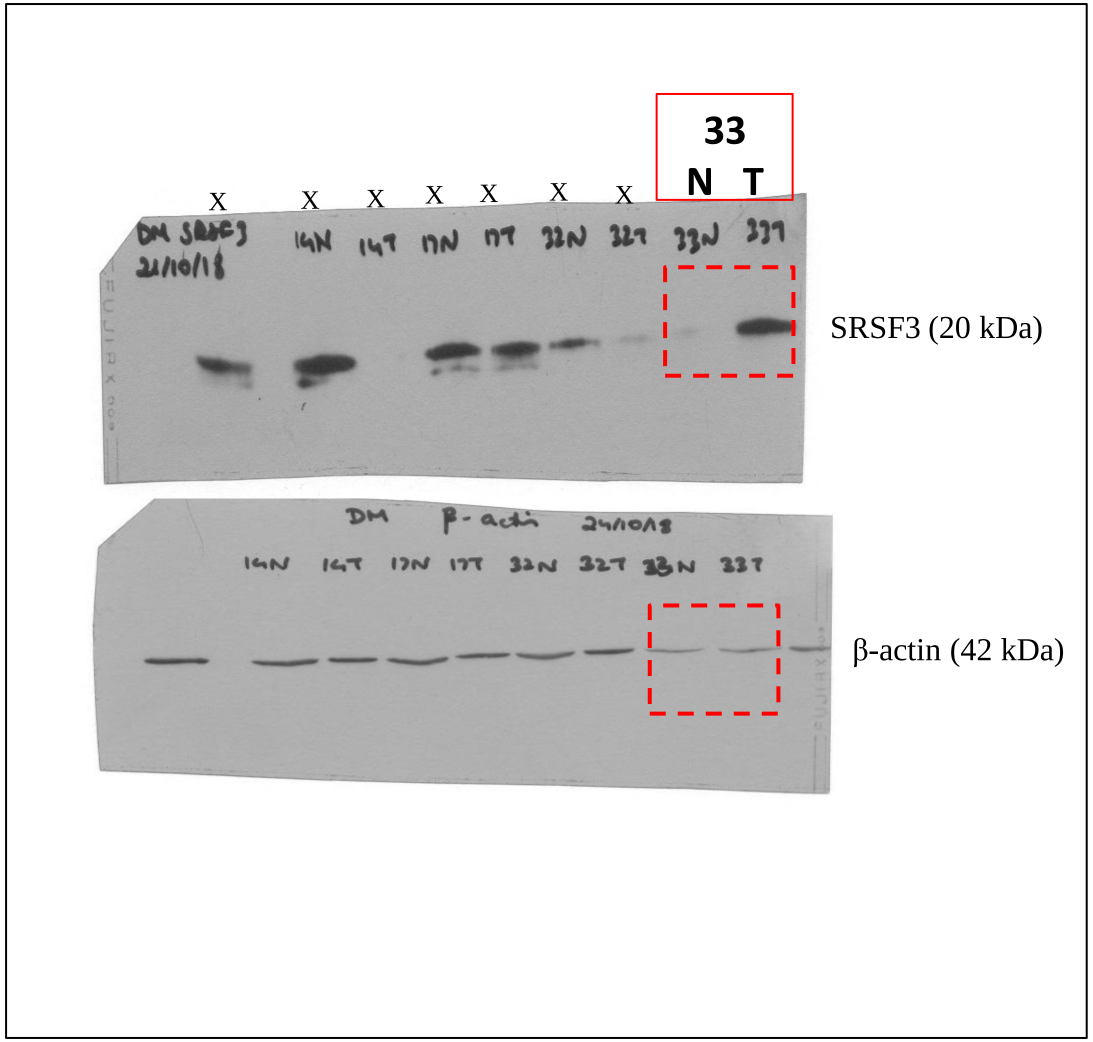

Full-length blots for S4 Fig.

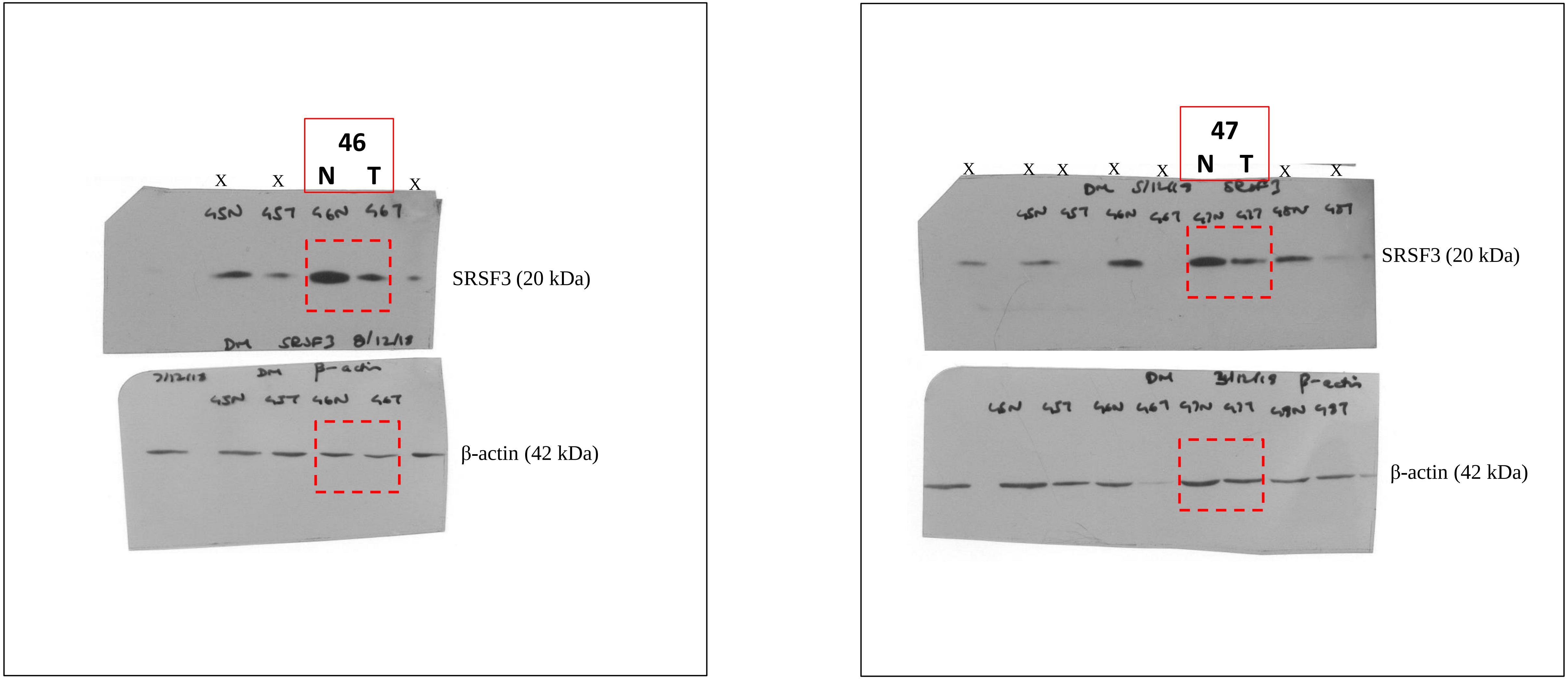

Full-length blots for S4 Fig.

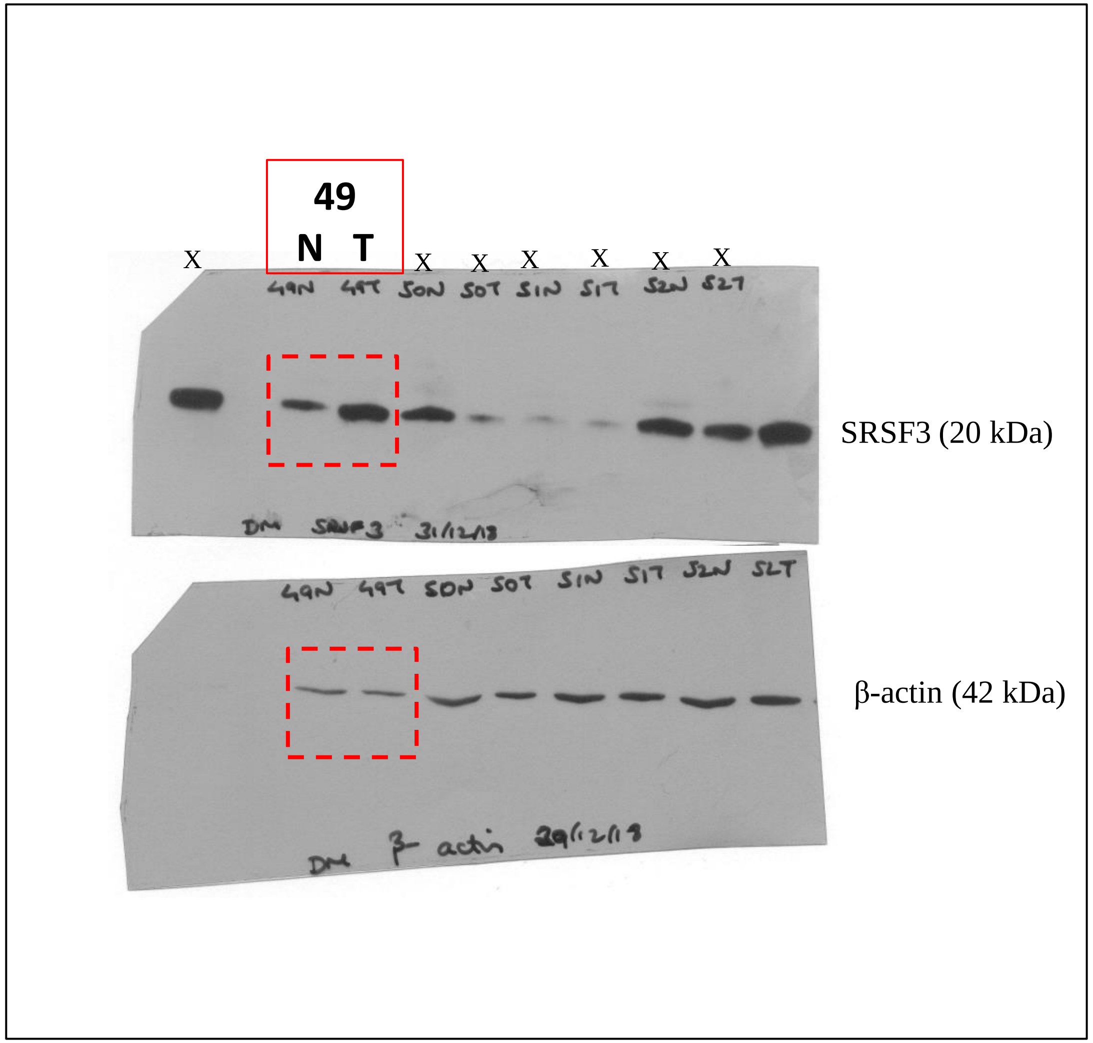

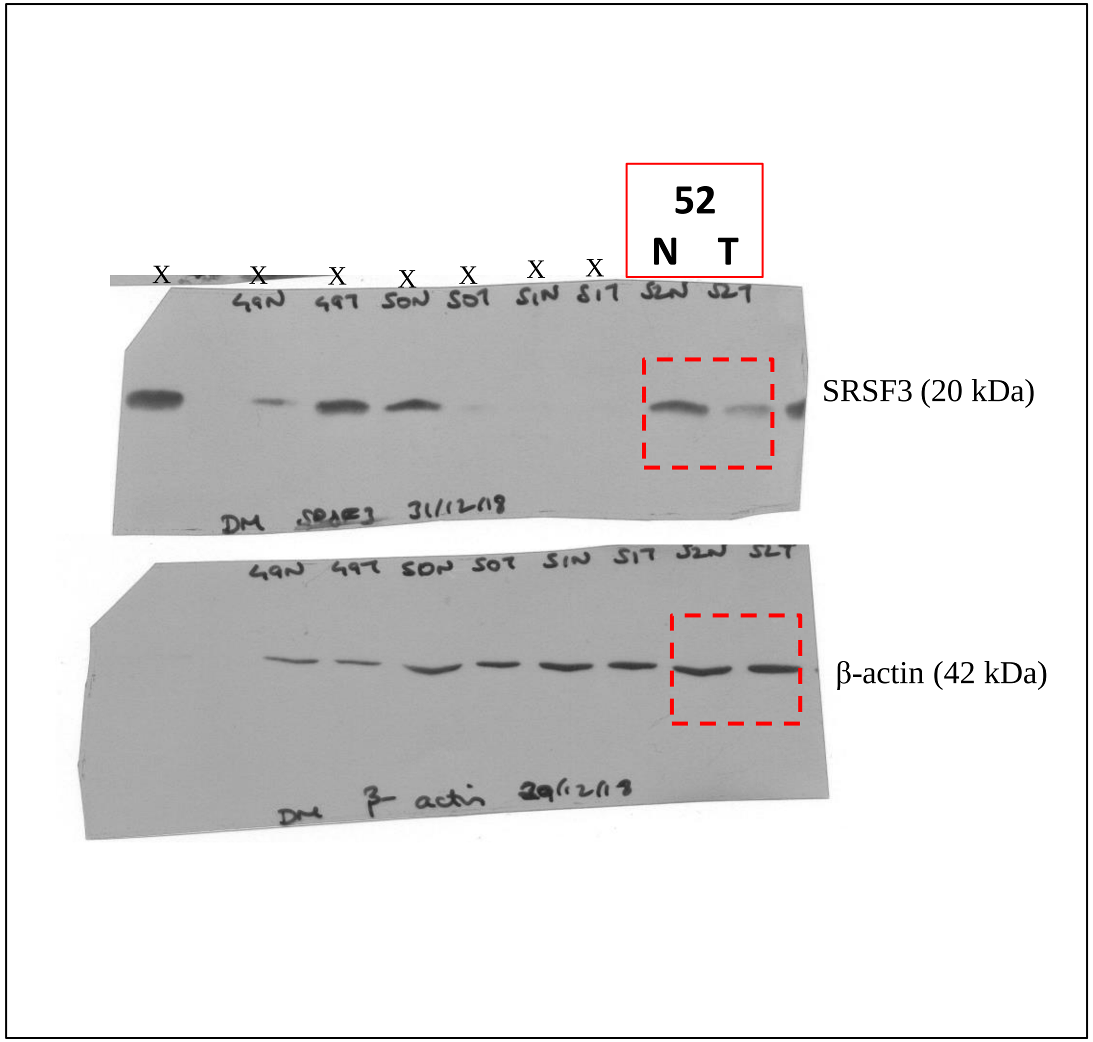

Full-length blots for S4 Fig.

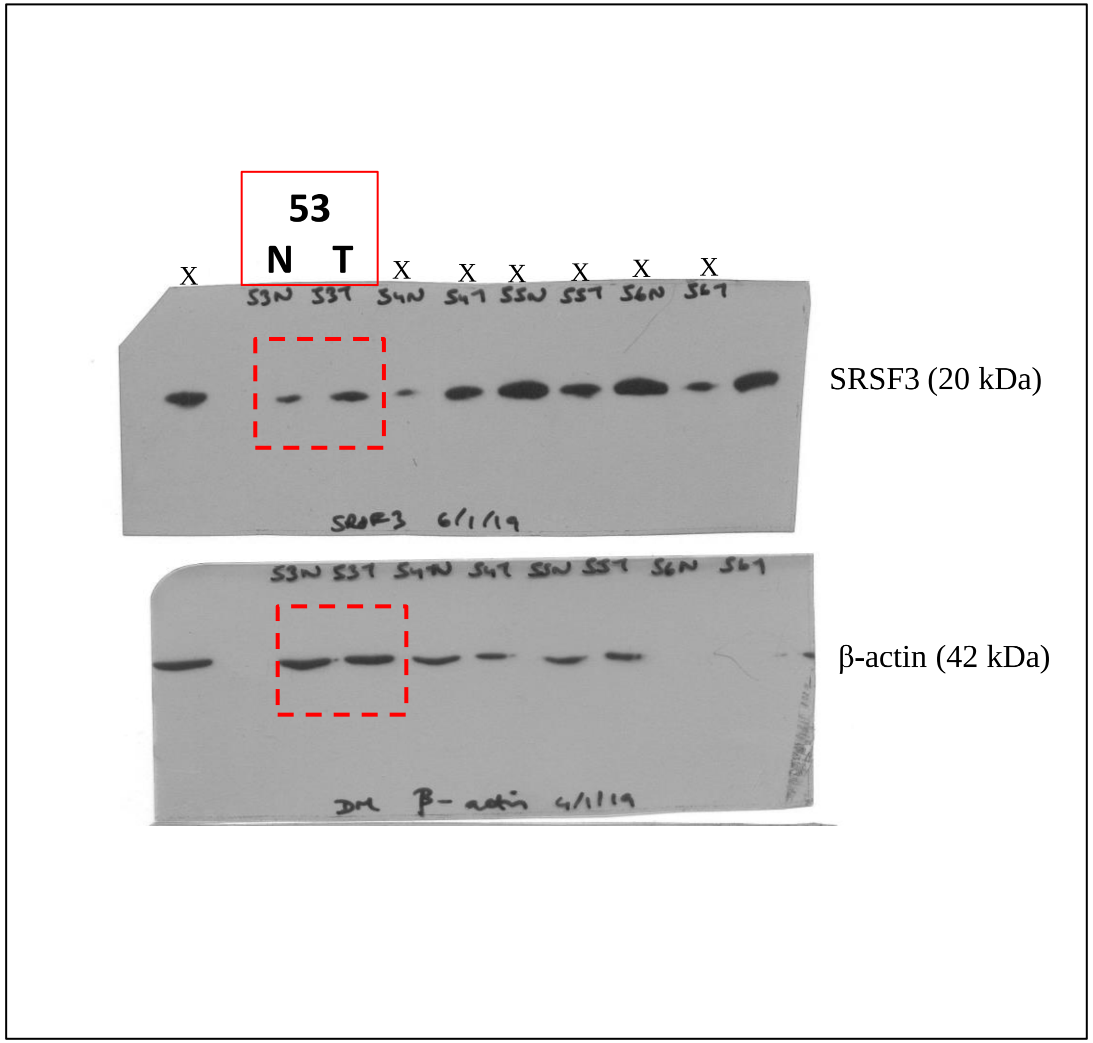

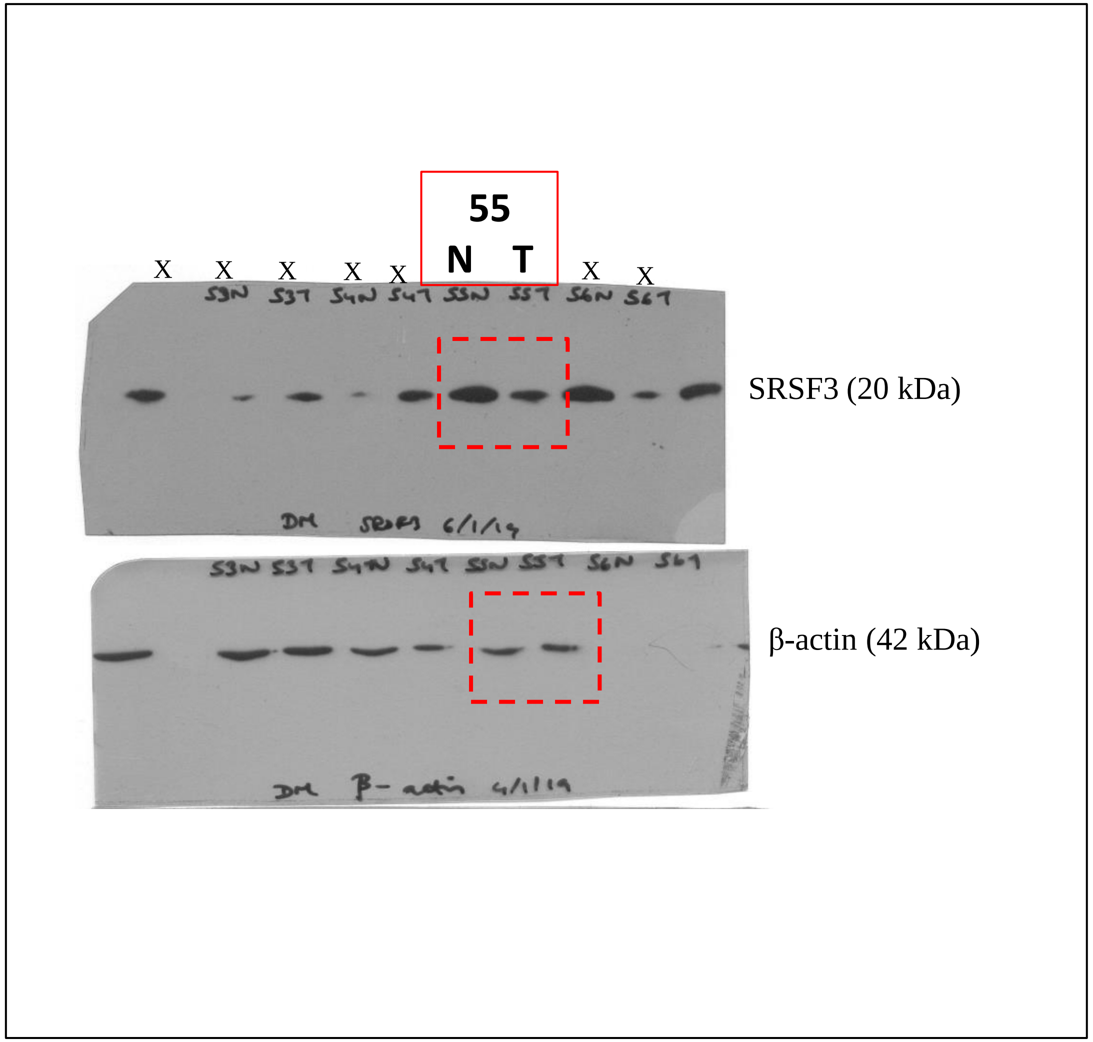

Full-length blots for S4 Fig.

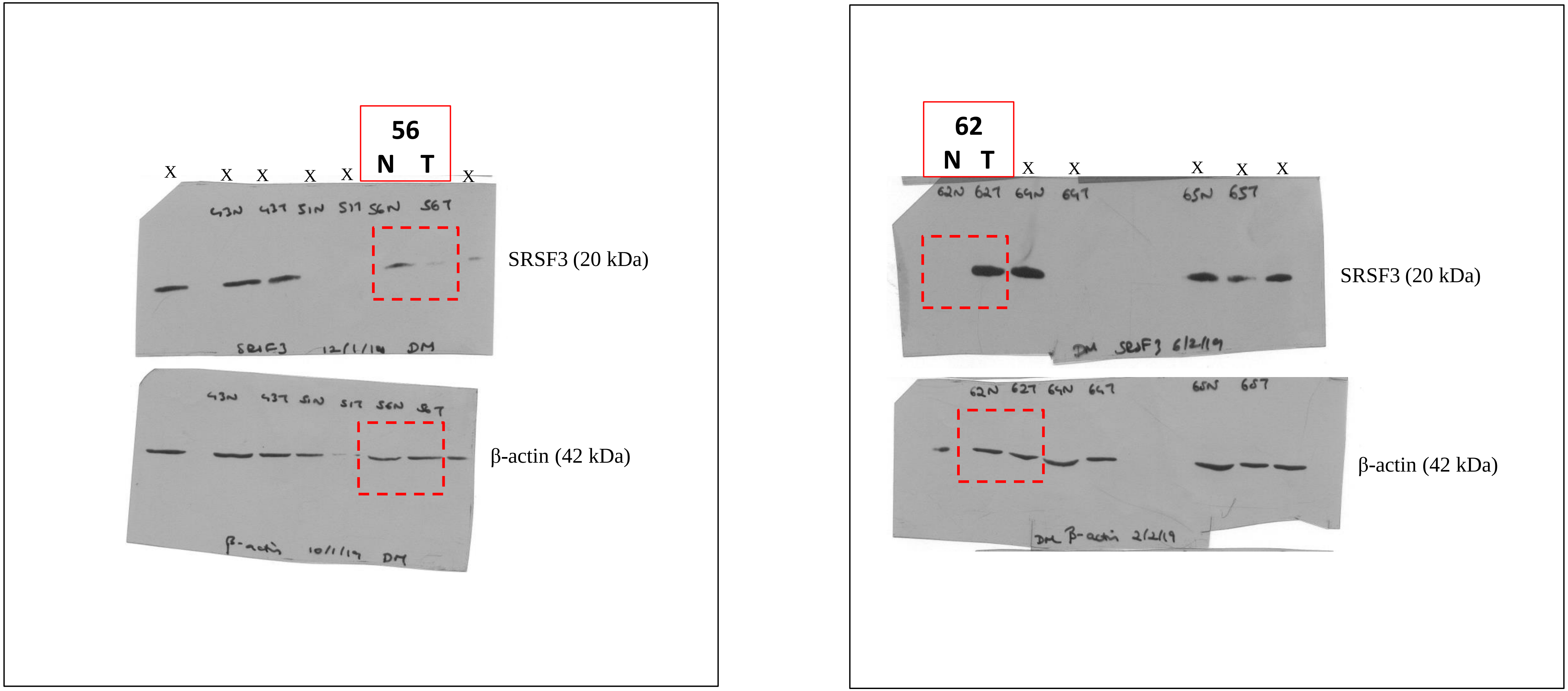

Full-length blots for S4 Fig.

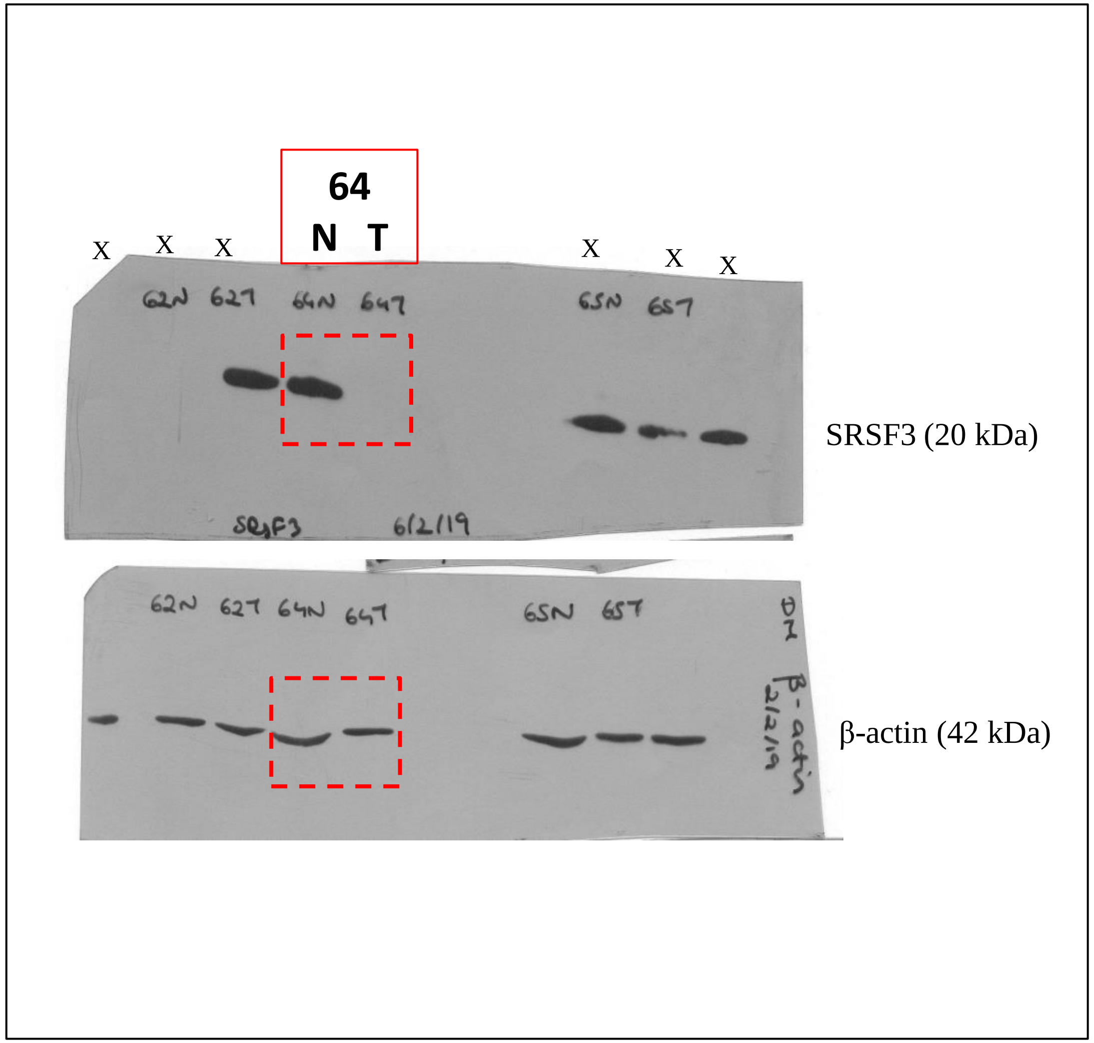

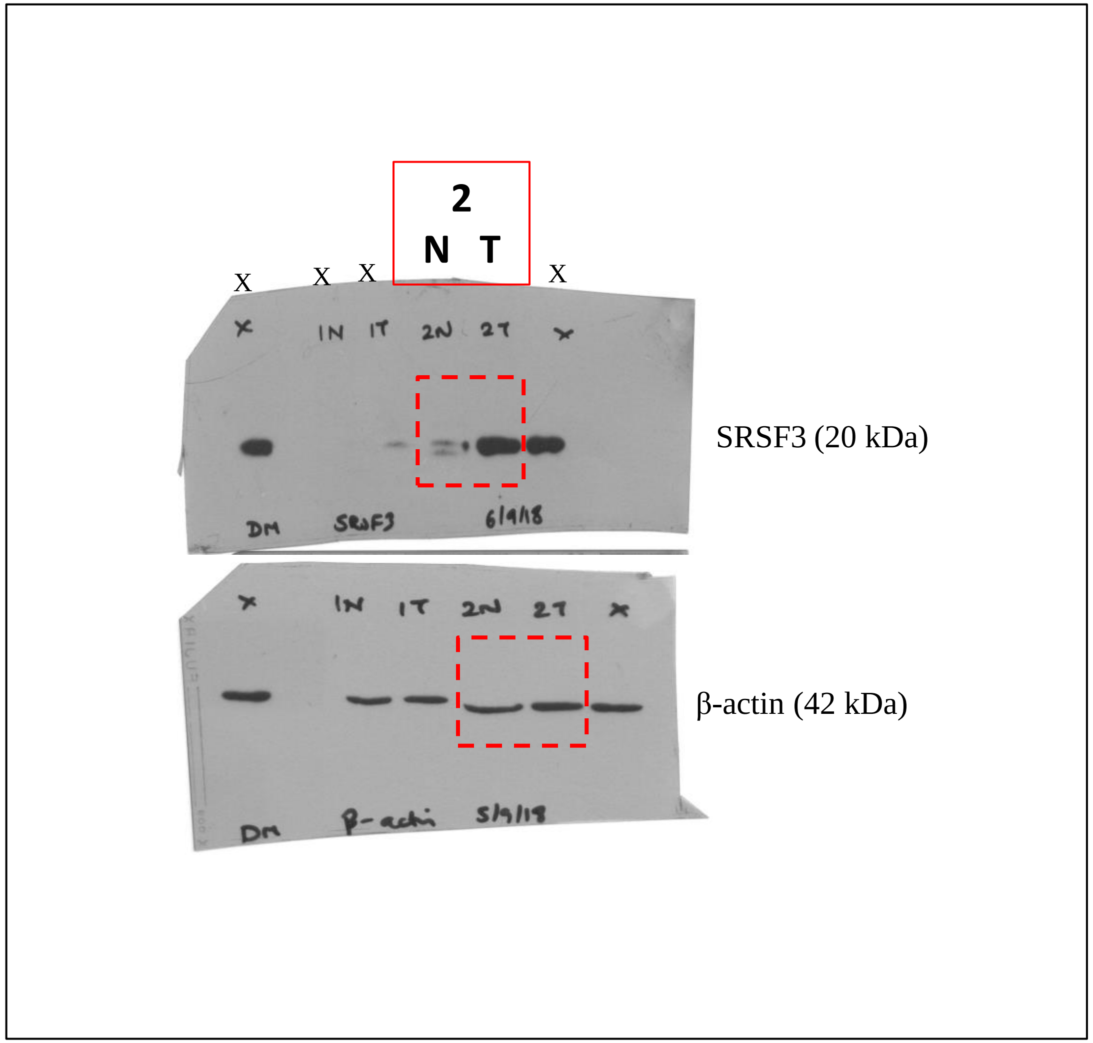

Full-length blots for S4 Fig.

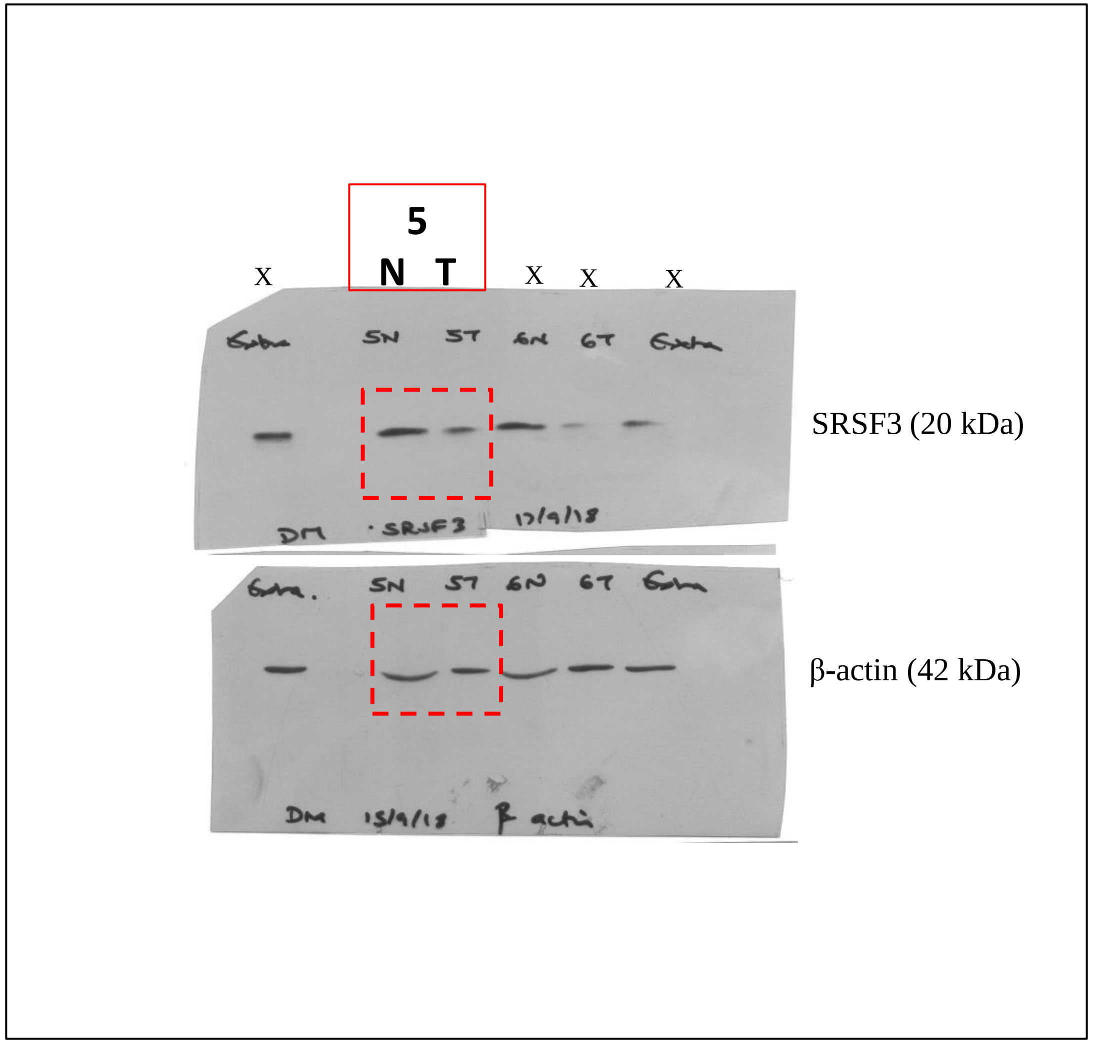

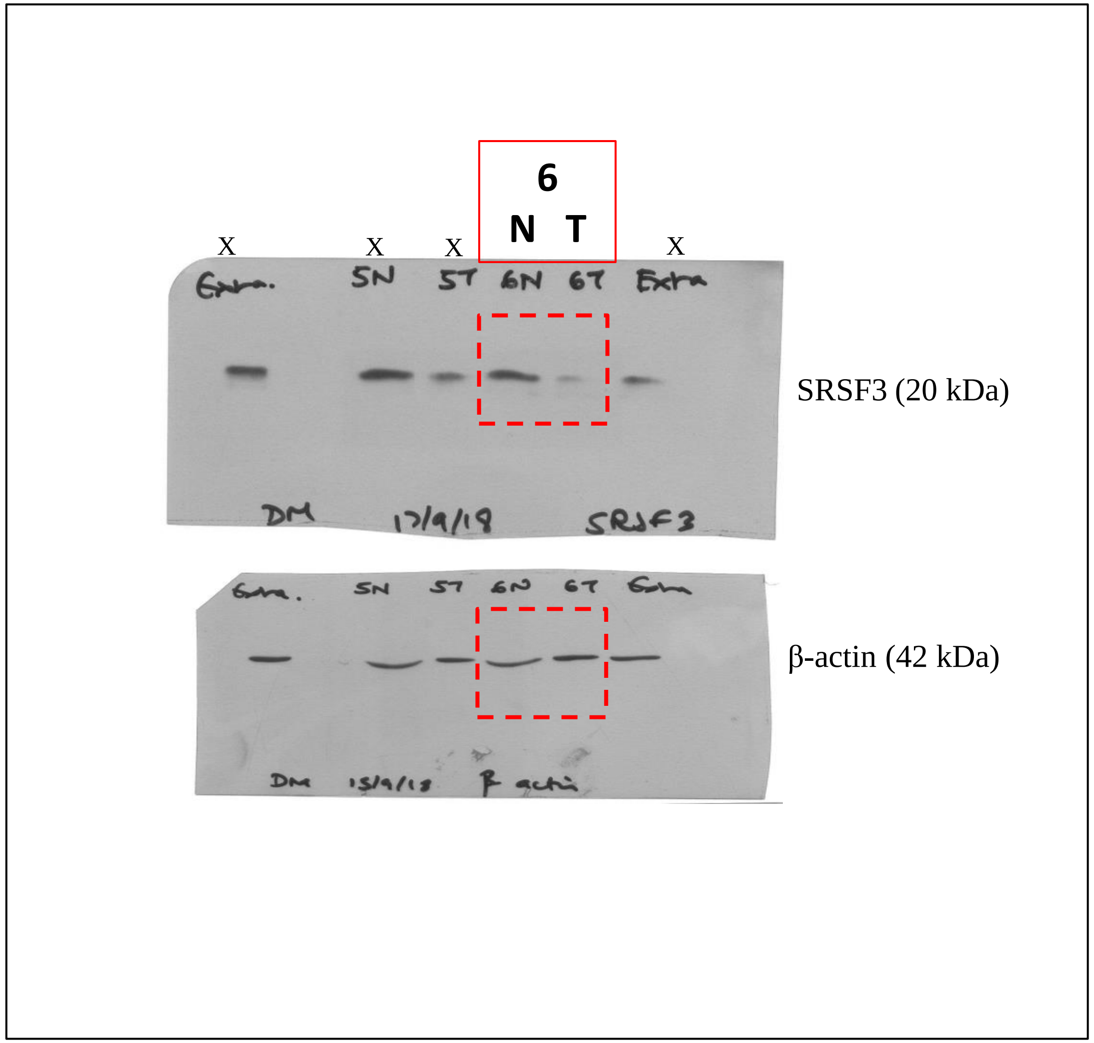

Full-length blots for S4 Fig.

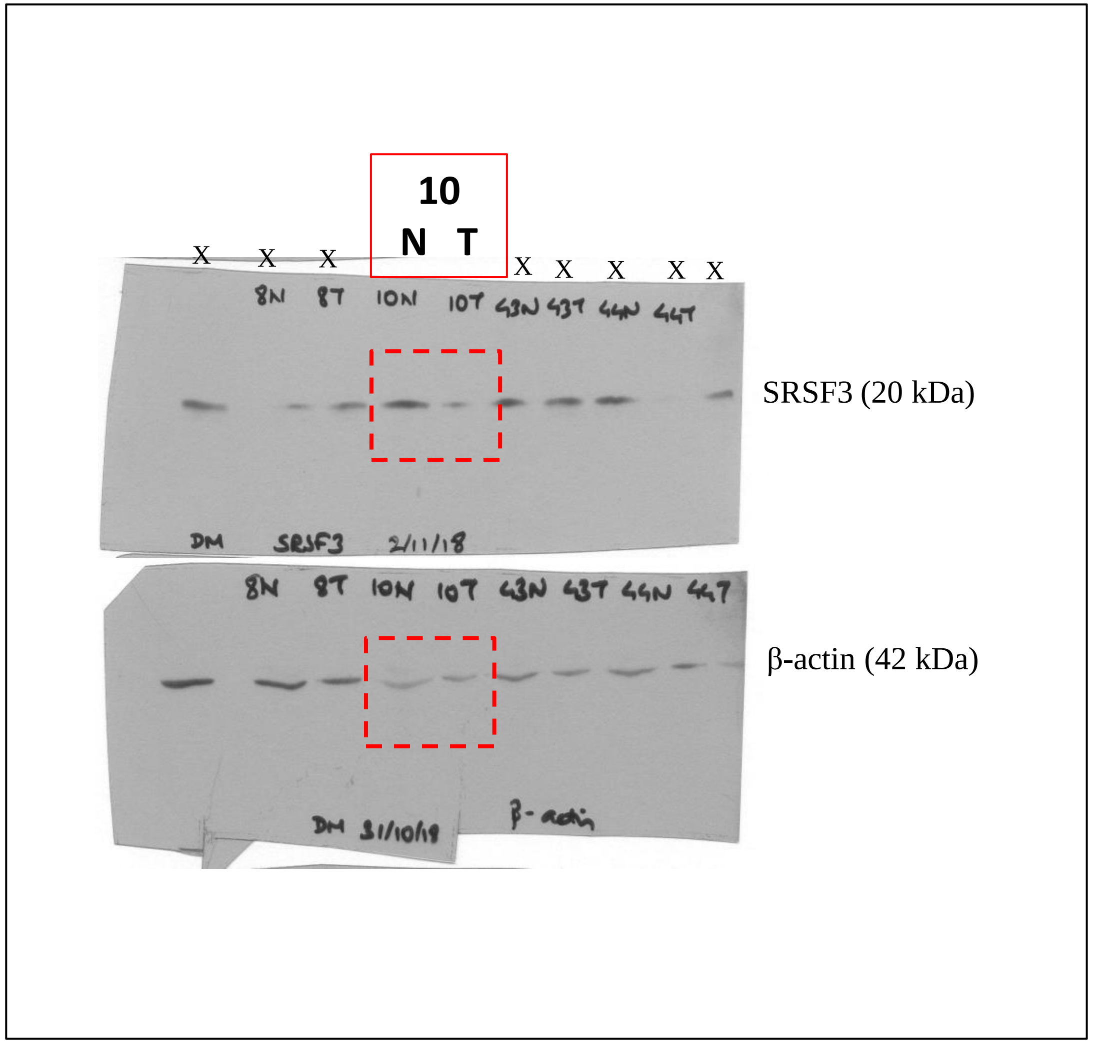

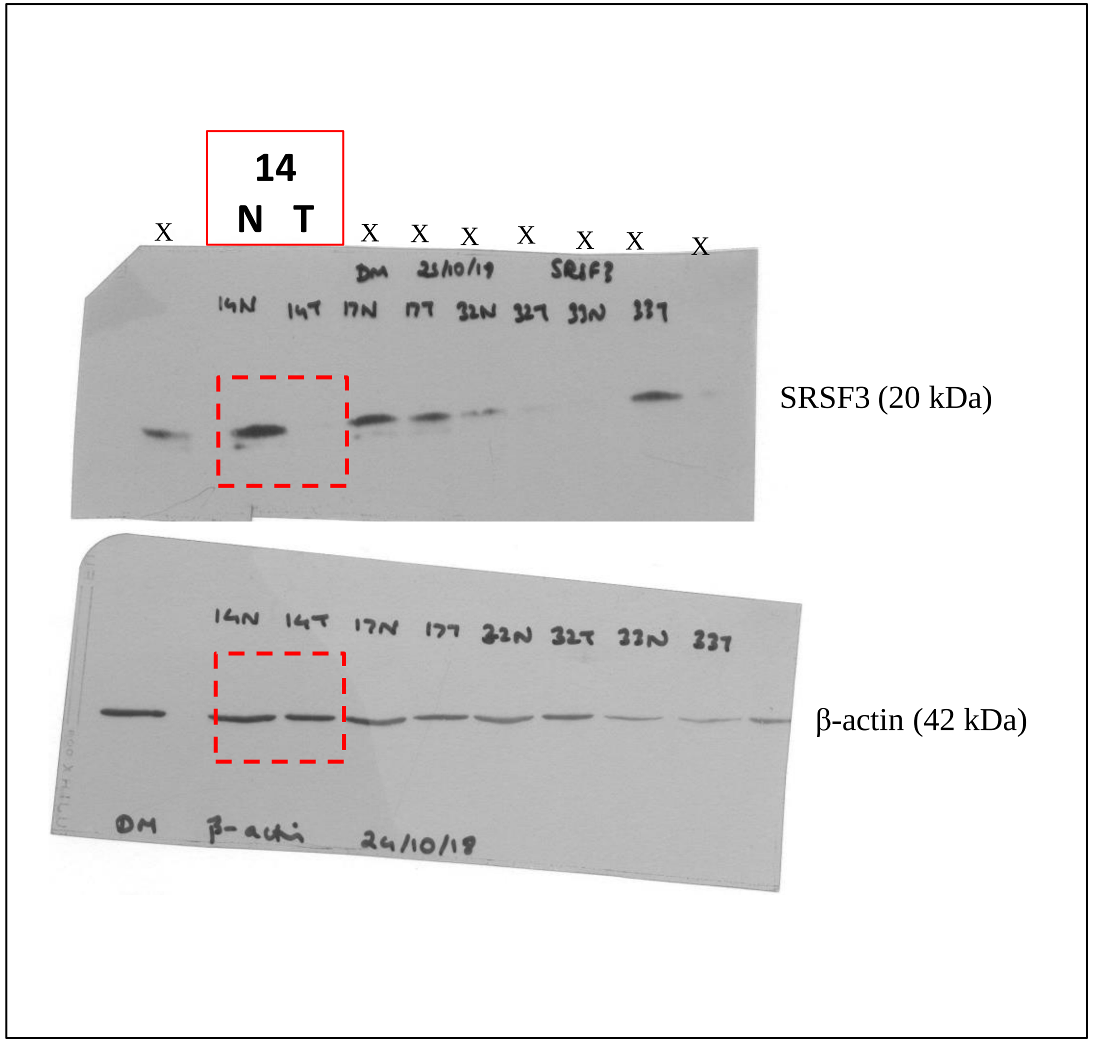

Full-length blots for S4 Fig.

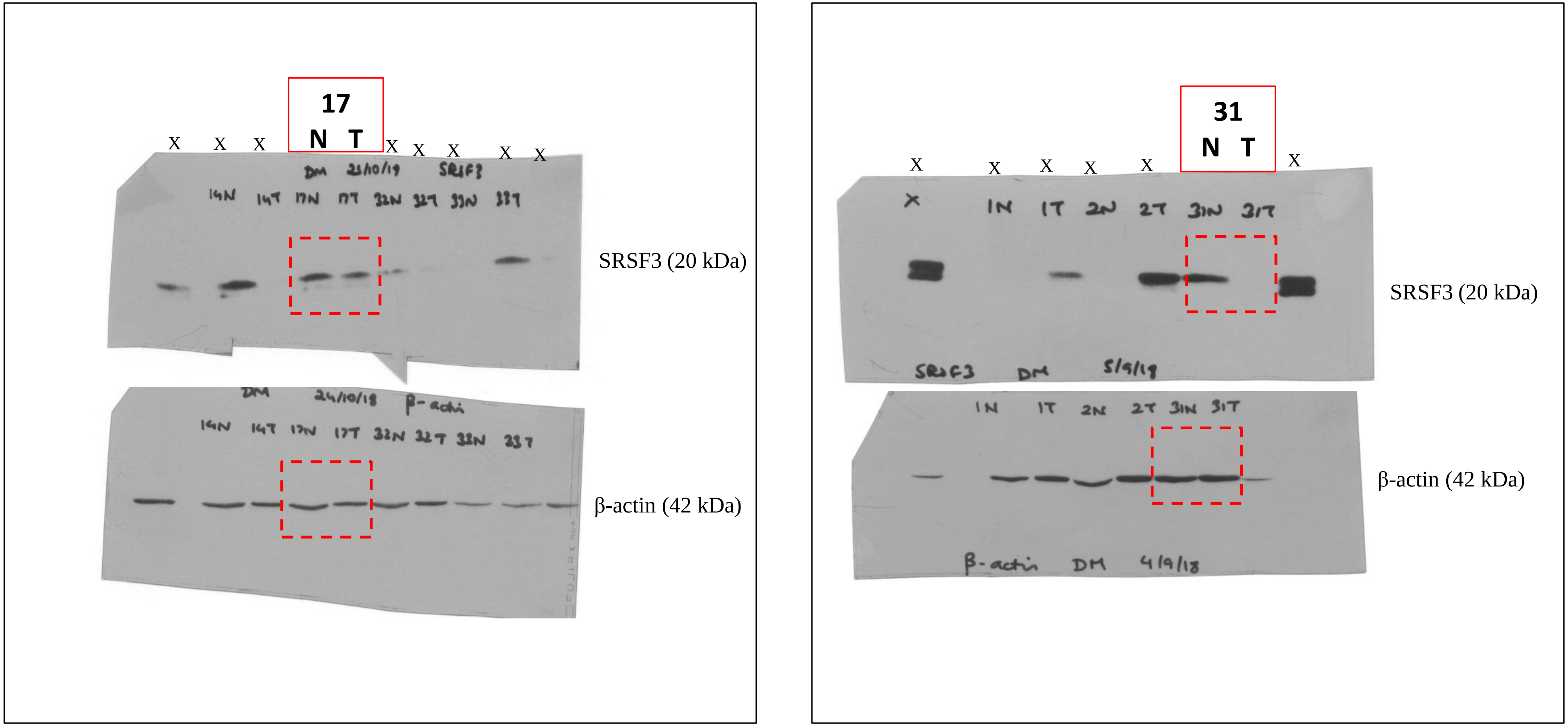

Full-length blots for S4 Fig.

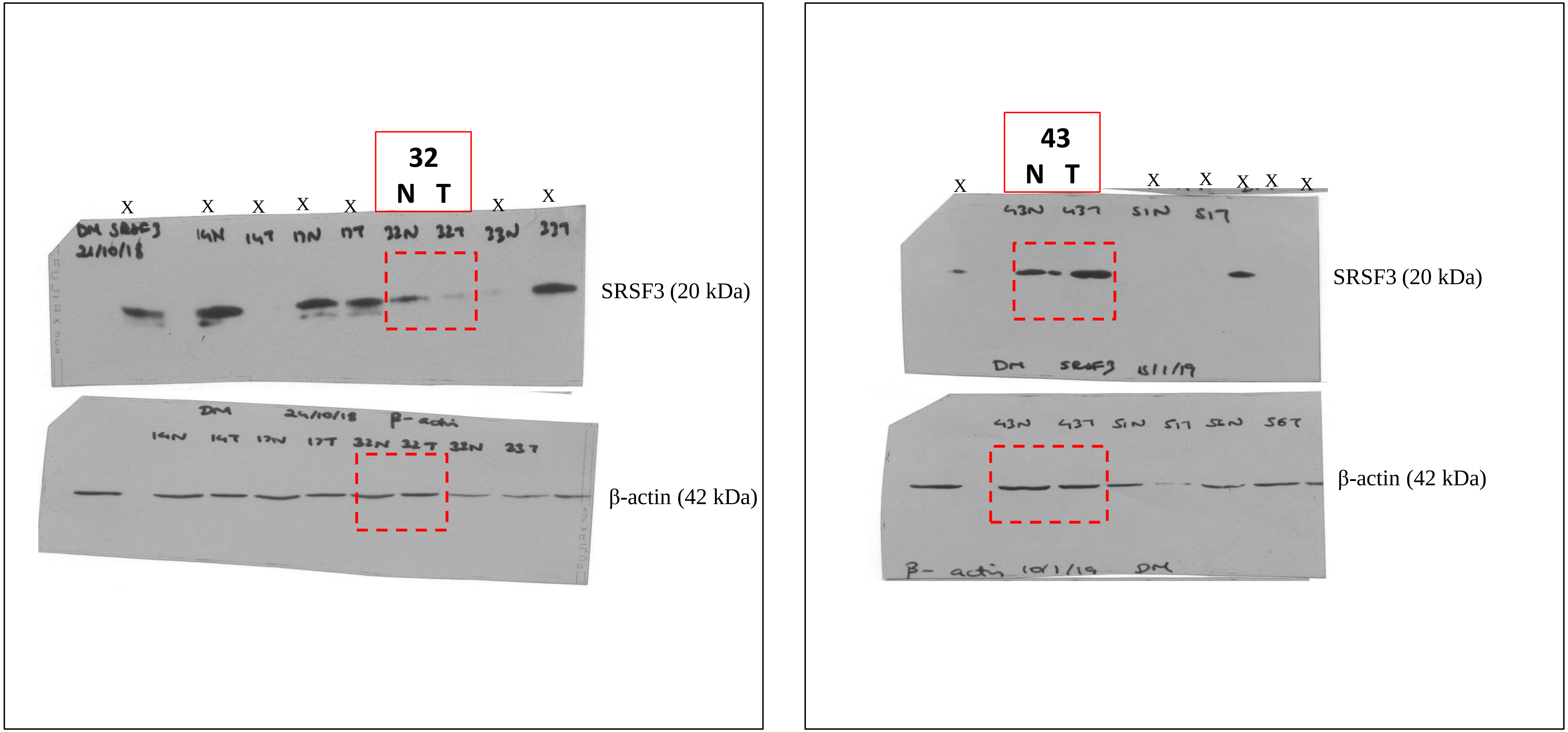

Full-length blots for S4 Fig.

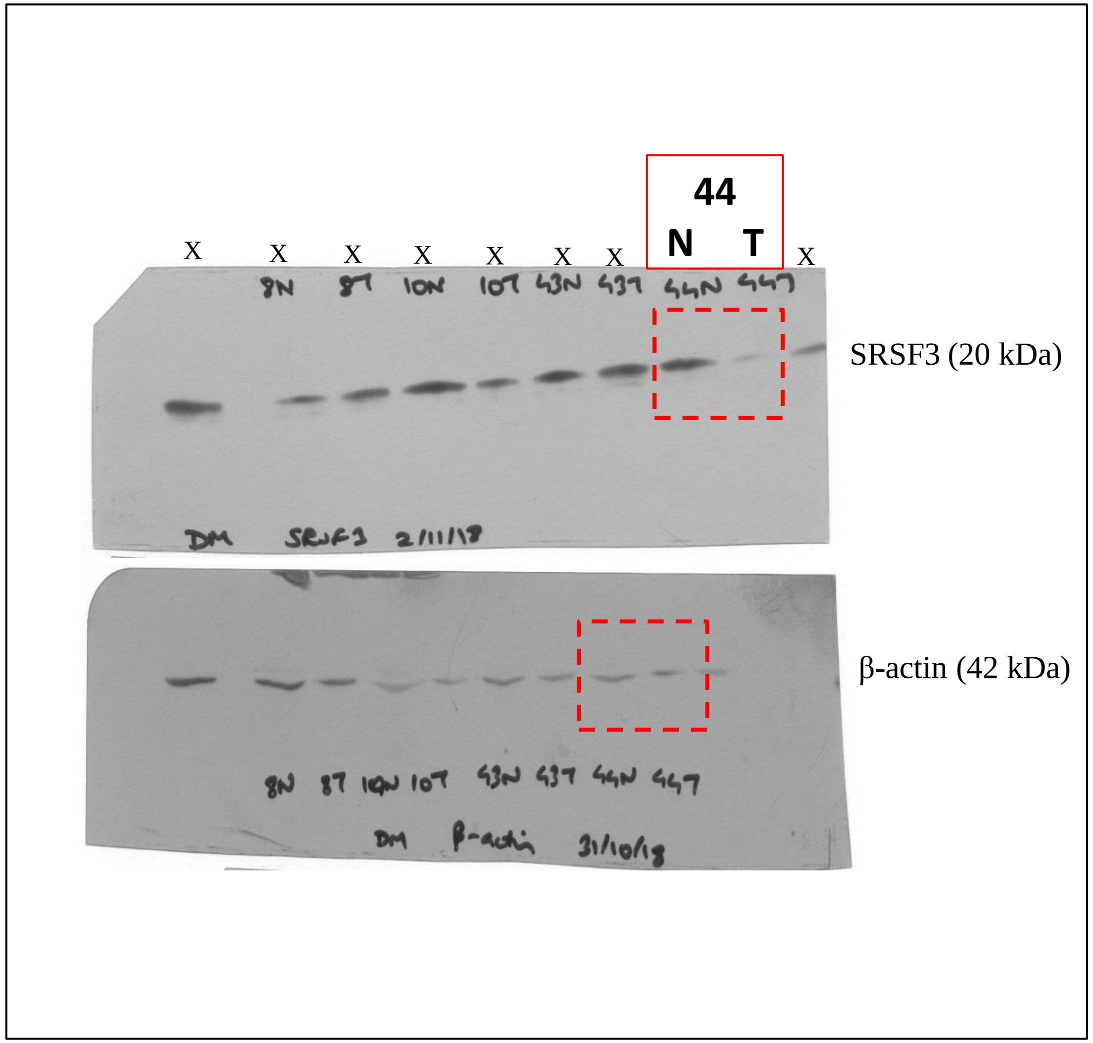

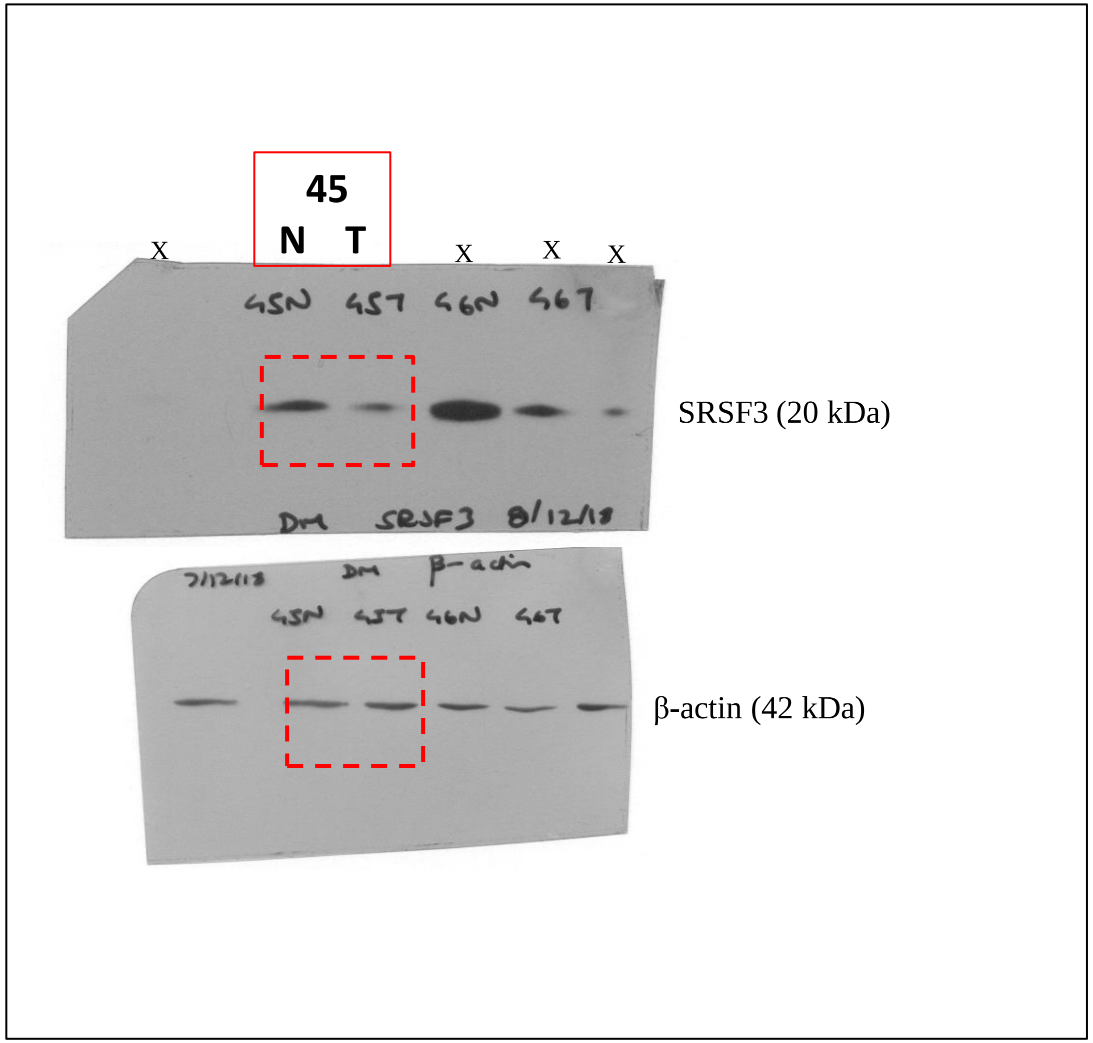

Full-length blots for S4 Fig.

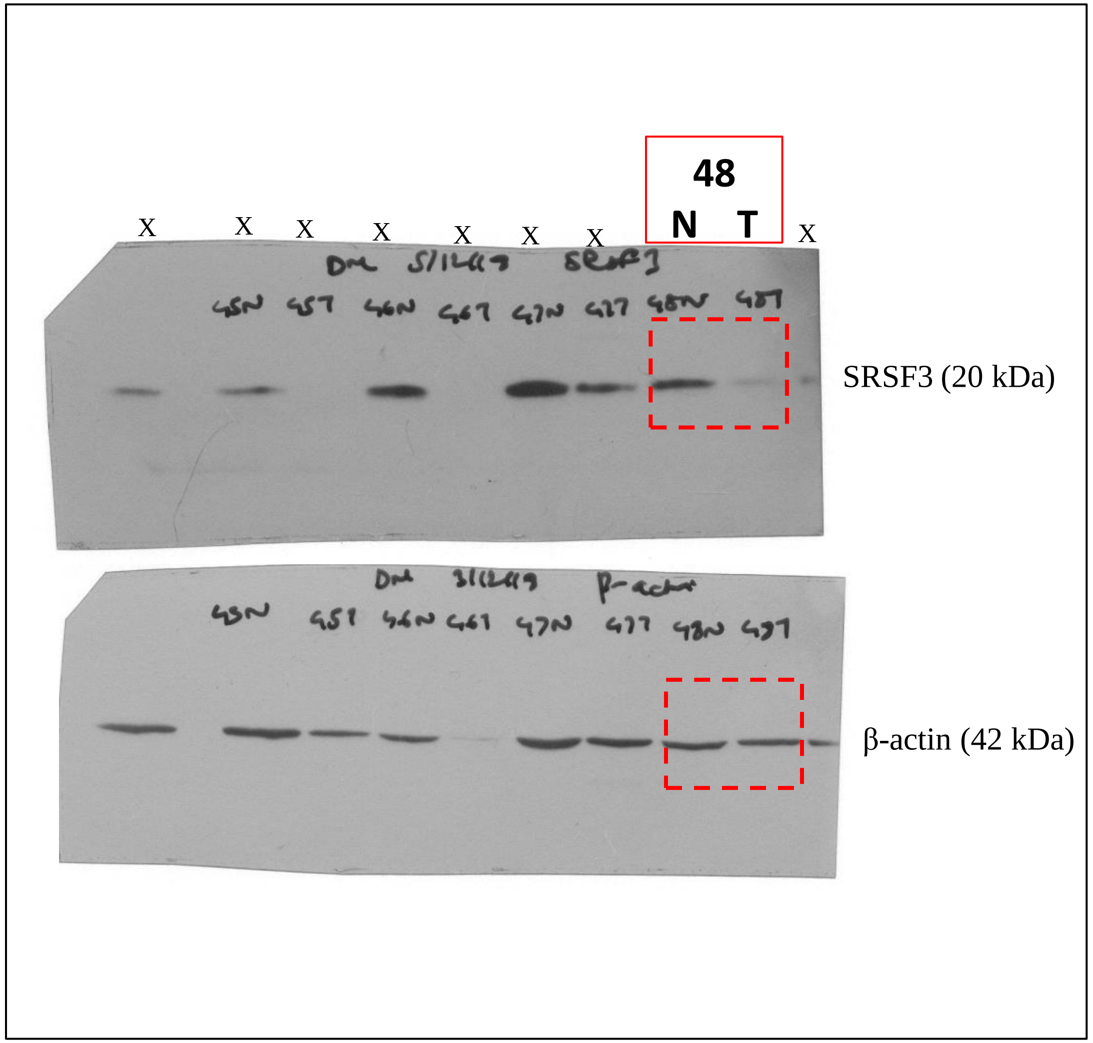

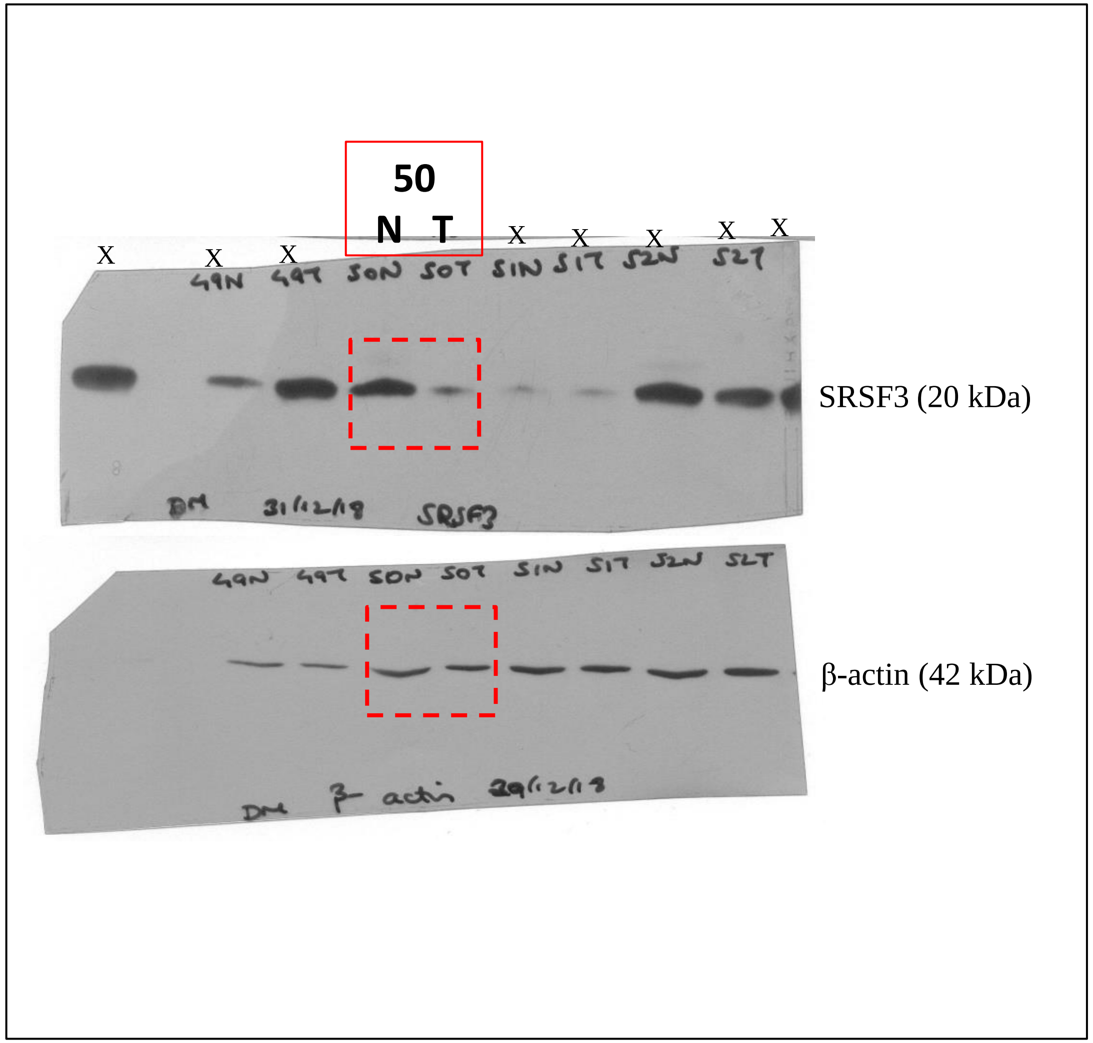

Full-length blots for S4 Fig.

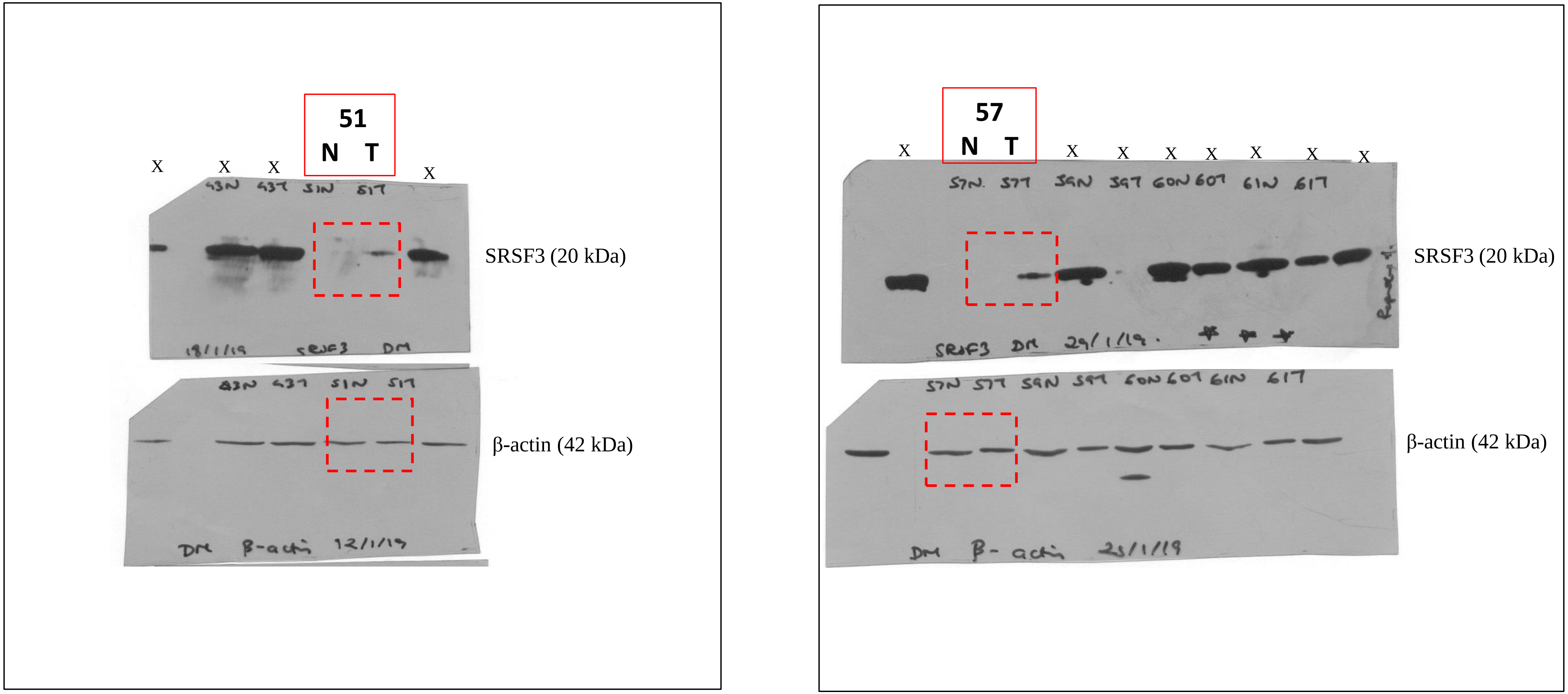

Full-length blots for S4 Fig.

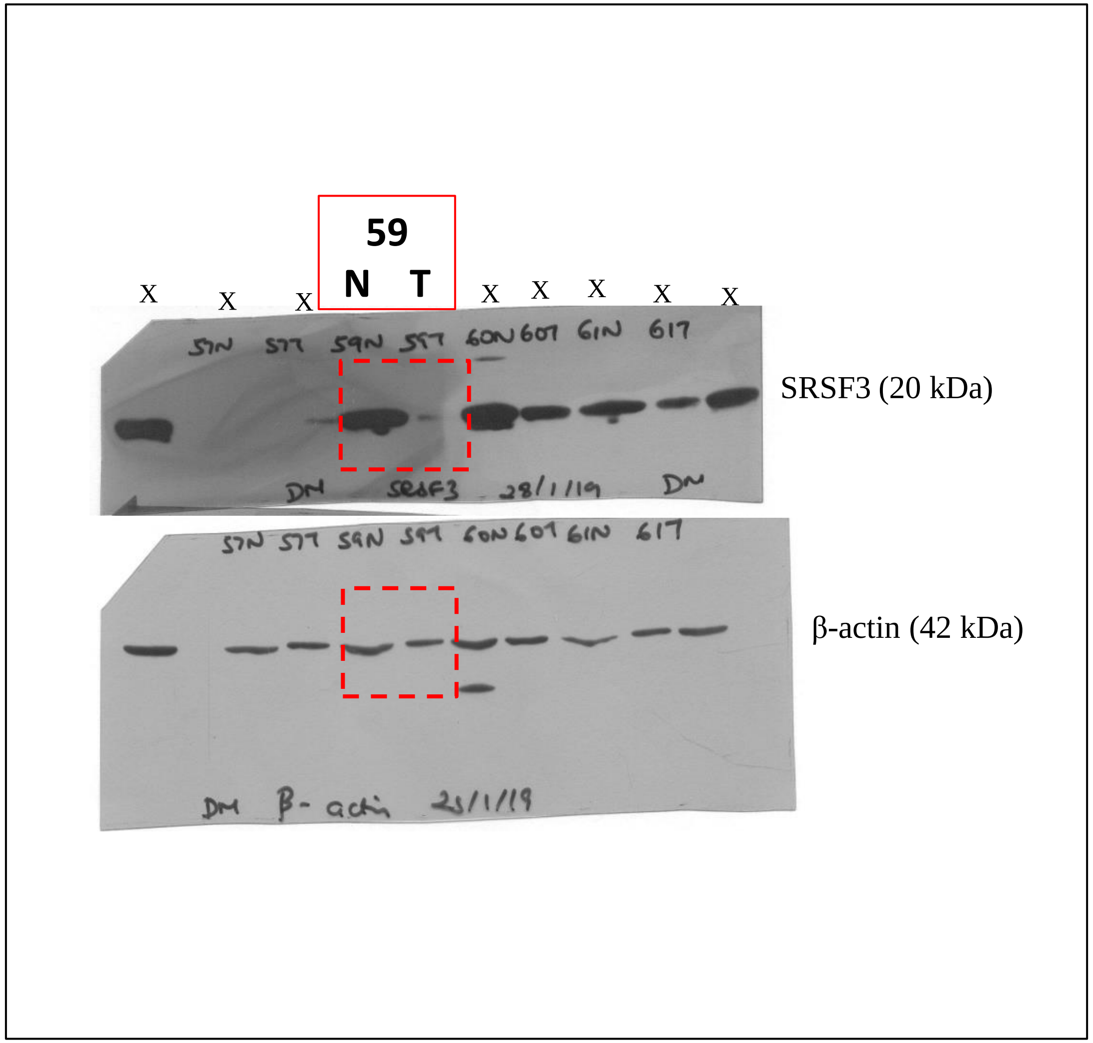

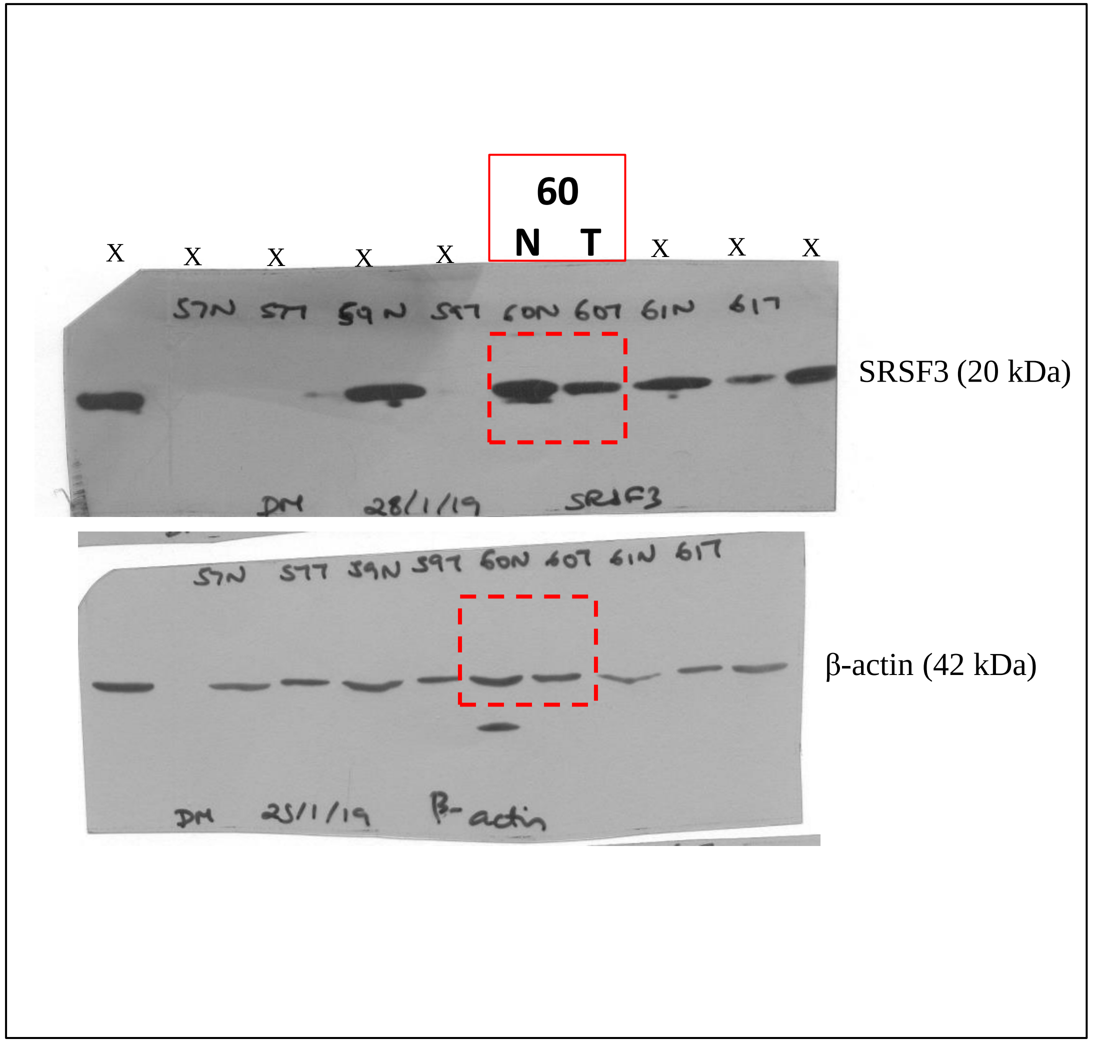

Full-length blots for S4 Fig.

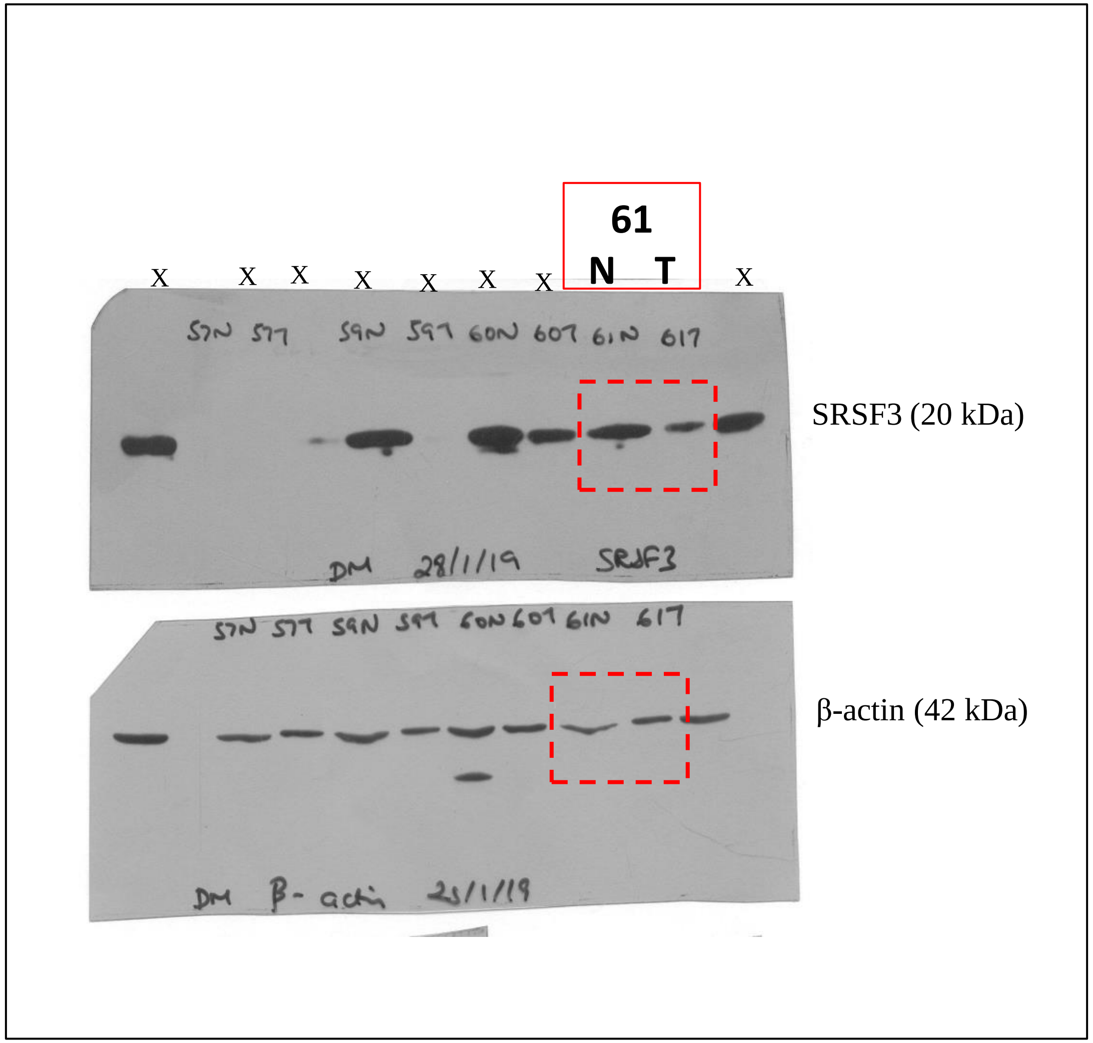

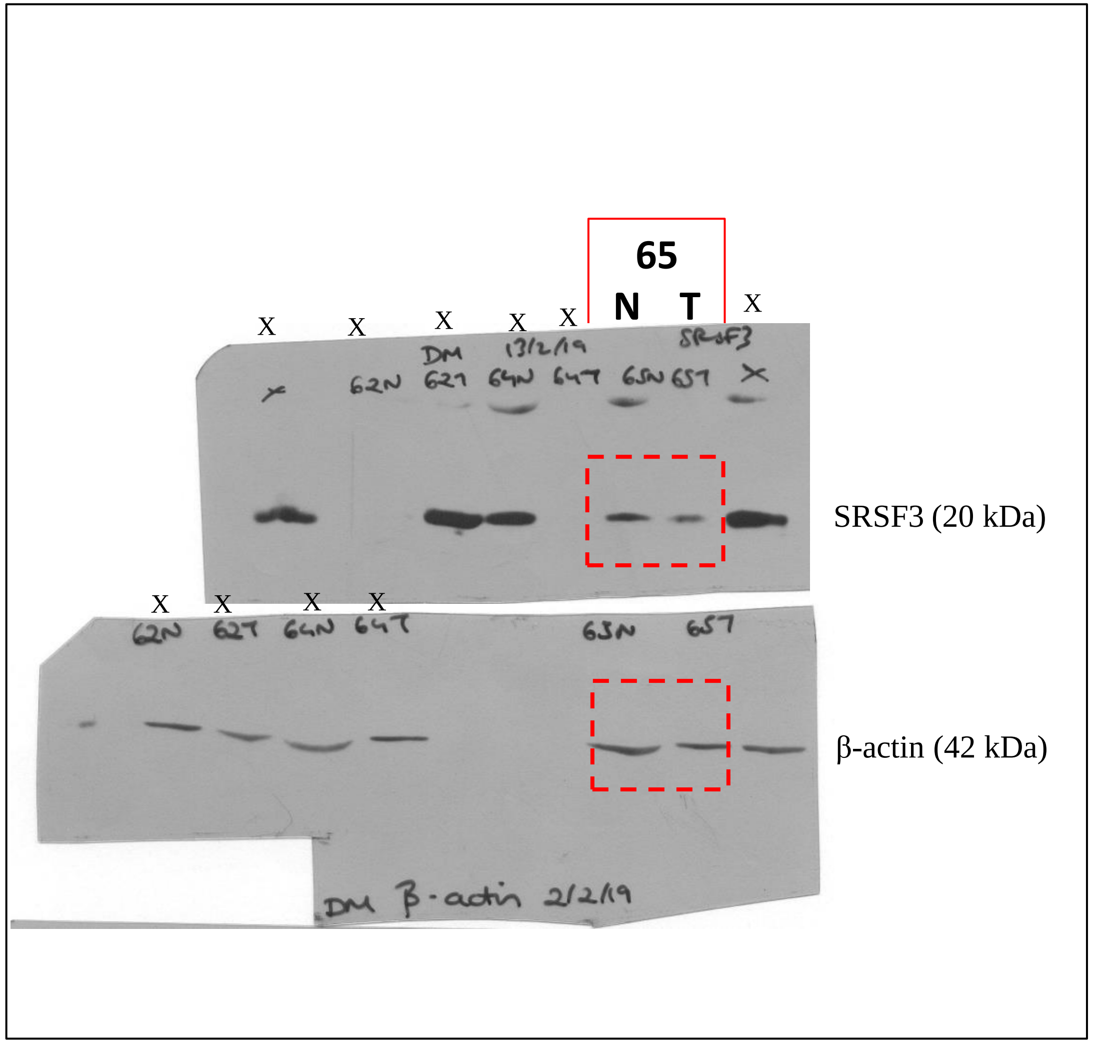

Full-length blots for S4 Fig.

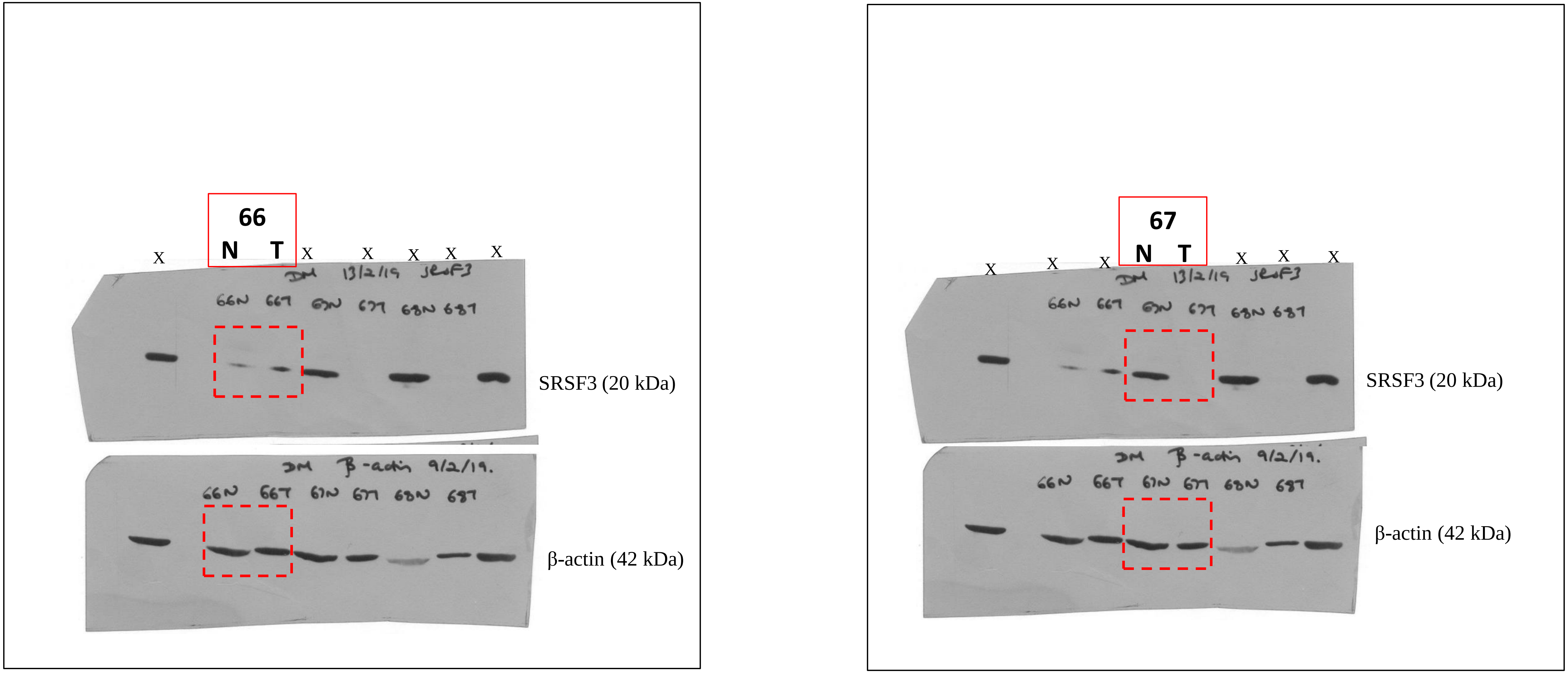

Full-length blots for S4 Fig.

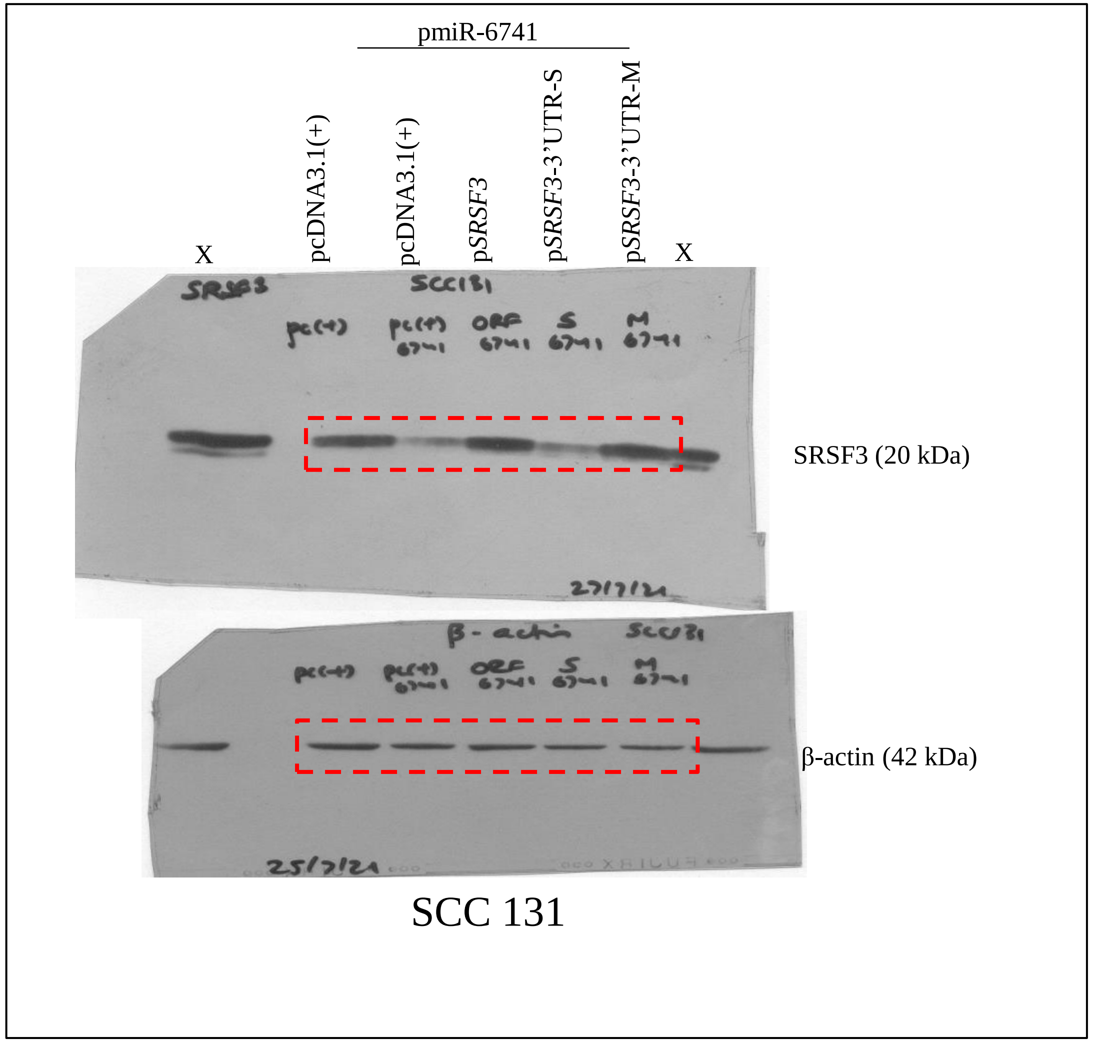

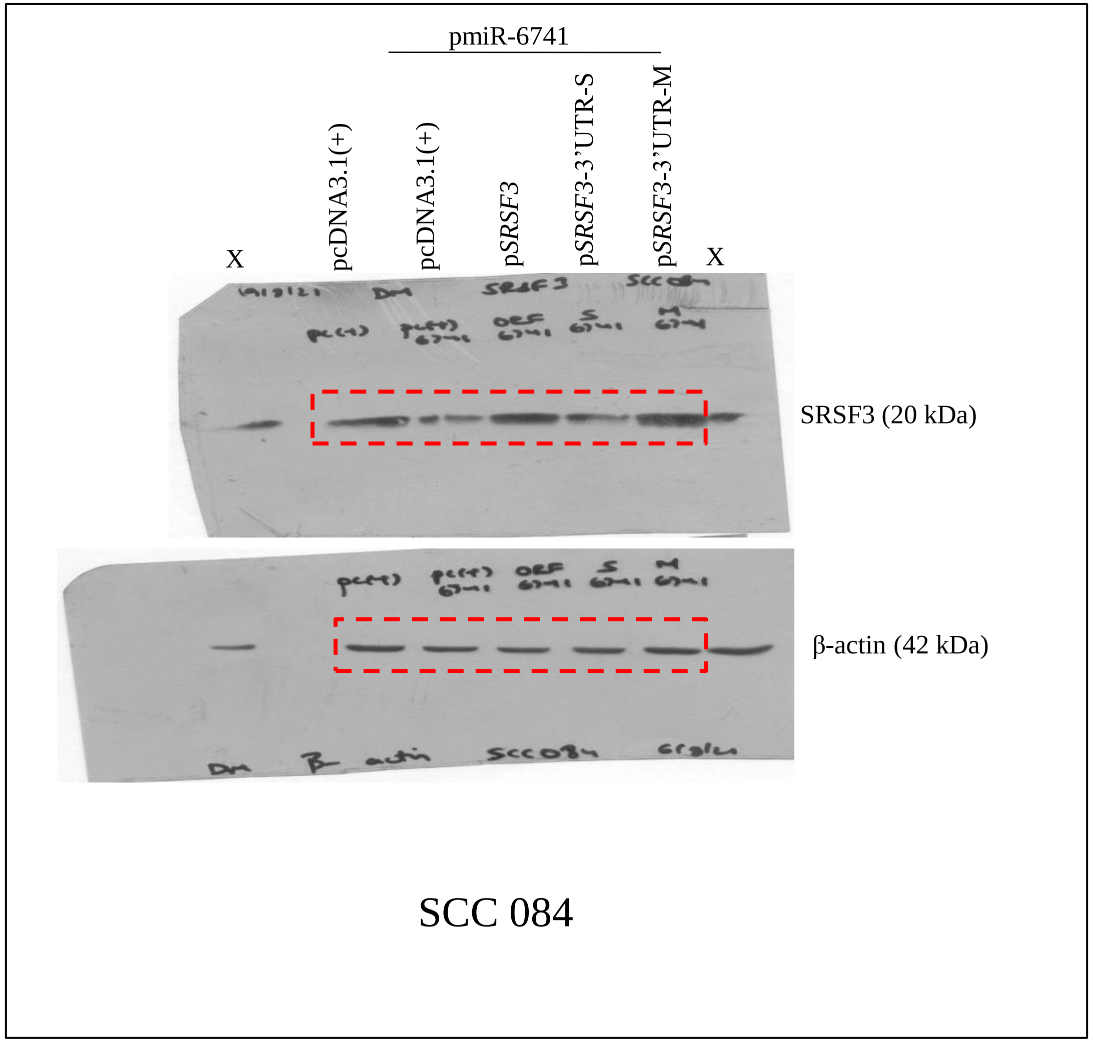

Full-length blots for S6 Fig.

A

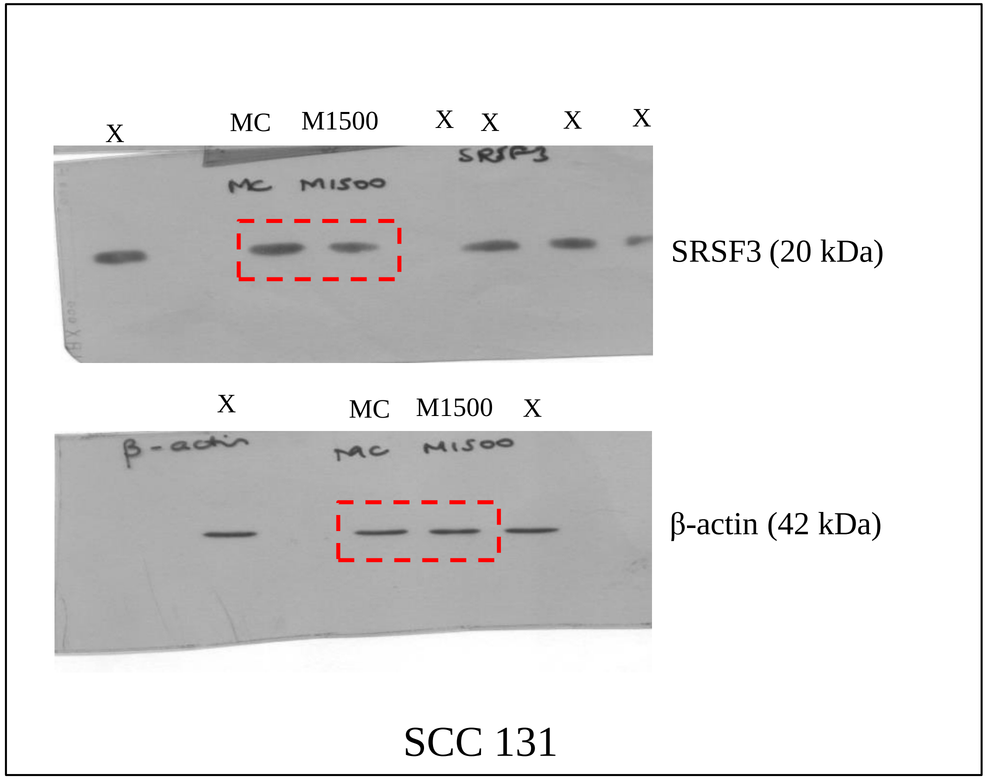

B

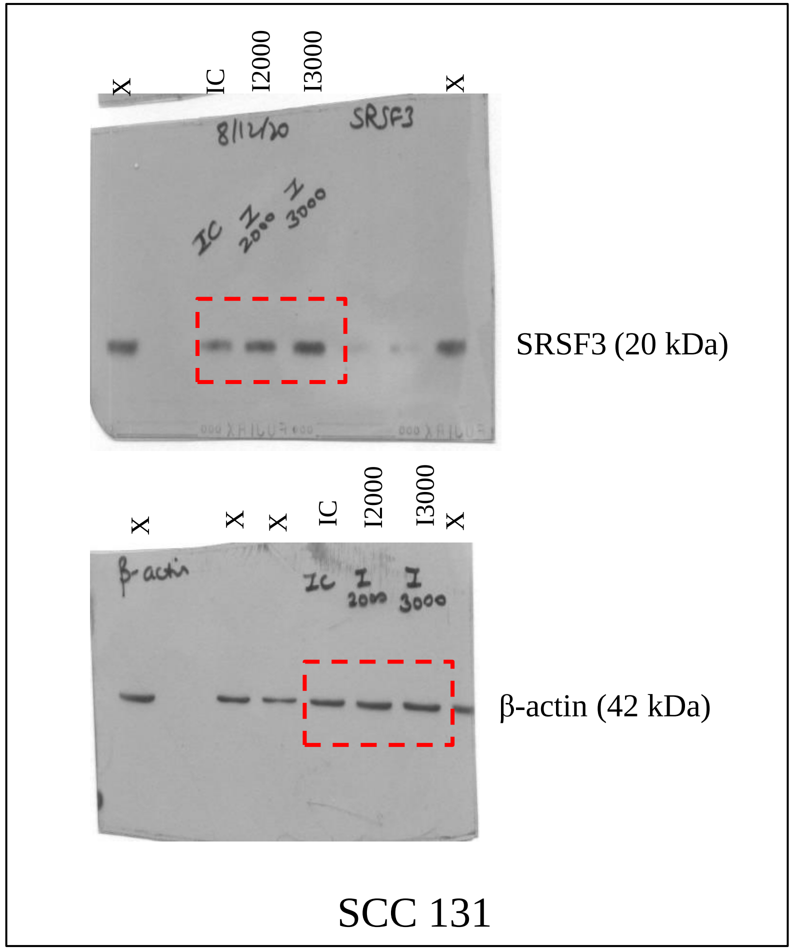

A) Full-length blots for S7A Fig. B) Full-length blots S7B Fig.

A

SCC 131

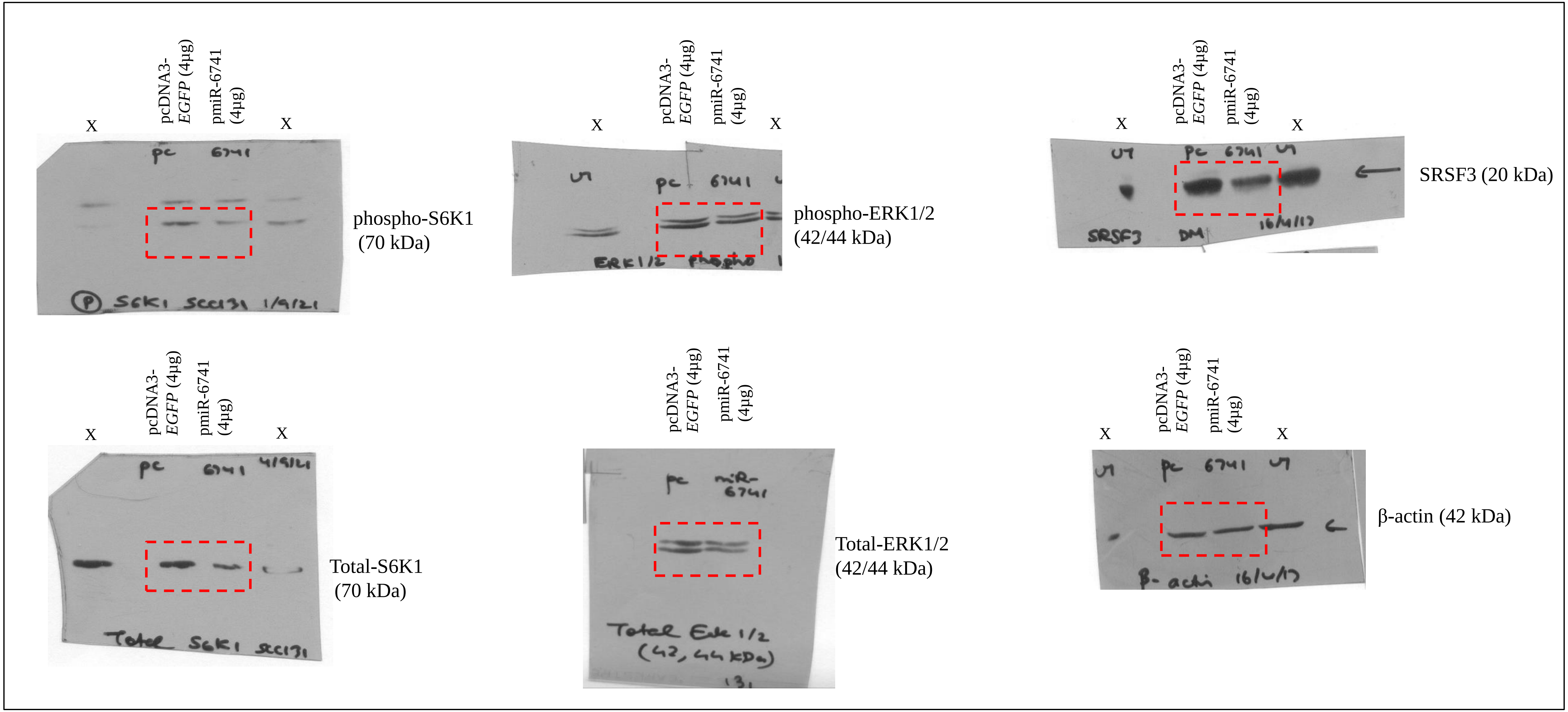

A) Full-length blots for S8A Fig. SCC131

A (continued)

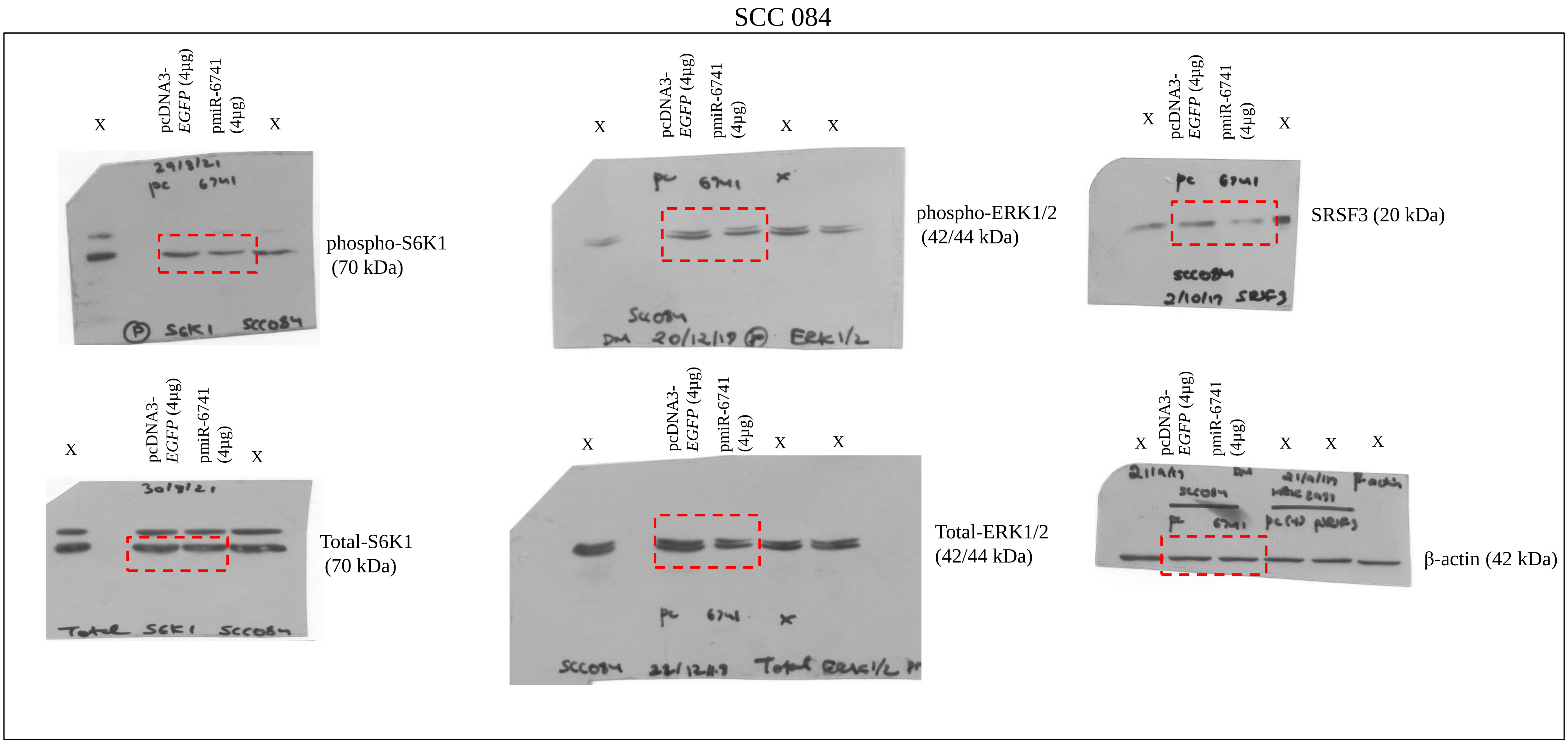

A) Full-length blots for S8A Fig. SCC084

B

SCC 131

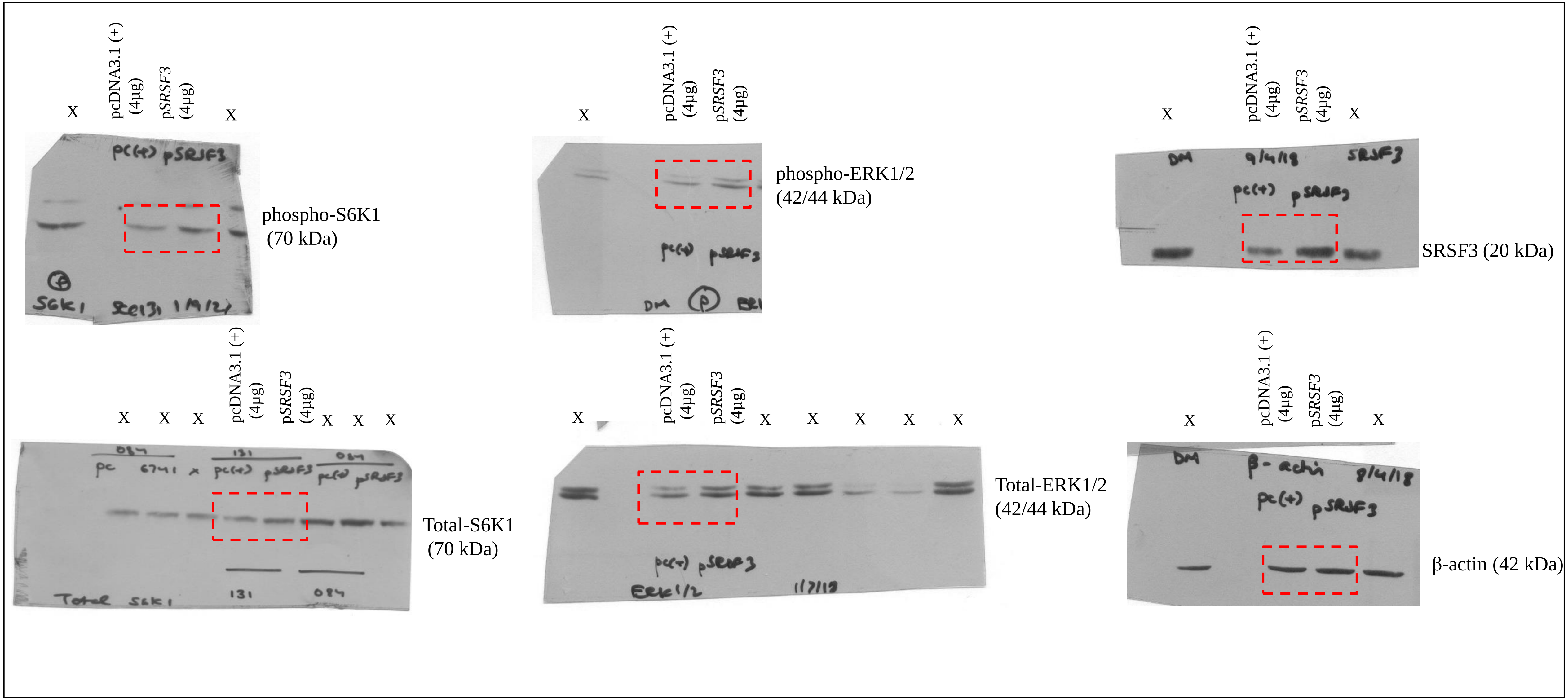

B) Full-length blots for S8B Fig. SCC131

B (Continued)

SCC 084

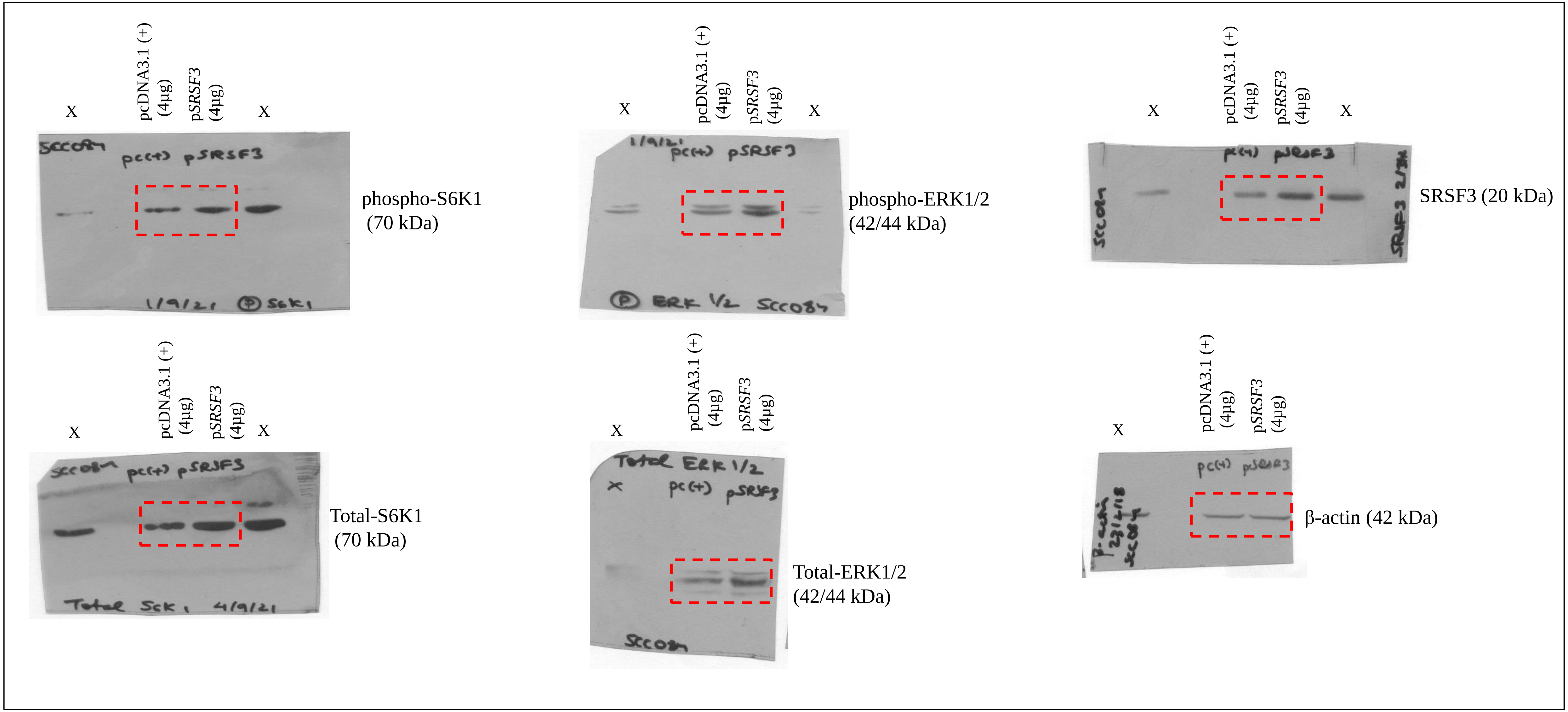

B) Full-length blots for S8B Fig. SCC084
